# Genome-wide survey, characterization, and expression analysis of bZIP transcription factors in *Chenopodium quinoa*

**DOI:** 10.1186/s12870-020-02620-z

**Published:** 2020-09-01

**Authors:** Feng Li, Jianxia Liu, Xuhu Guo, Lili Yin, Hongli Zhang, Riyu Wen

**Affiliations:** 1 College of Life Science, Shanxi Datong University, Datong, 037009 People’s Republic of China; 2 Research and Development Center of Agricultural Facility Technology, Shanxi Datong University, Datong, 037009 People’s Republic of China; 3 Maize Research Institute, Shanxi Academy of Agricultural Sciences, Xinzhou, 034000 People’s Republic of China

**Keywords:** Quinoa (*Chenopodium quinoa*), bZIP transcription factor family, Phylogenetic classification, Evolutionary analysis, Gene expression patterns

## Abstract

**Background:**

*Chenopodium quinoa* Willd. (quinoa) is a pseudocereal crop of the Amaranthaceae family and represents a promising species with the nutritional content and high tolerance to stressful environments, such as soils affected by high salinity. The basic leucine zipper (bZIP) transcription factor represents exclusively in eukaryotes and can be related to many biological processes. So far, the genomes of quinoa and 3 other Amaranthaceae crops (*Spinacia oleracea*, *Beta vulgaris*, and *Amaranthus hypochondriacus*) have been fully sequenced. However, information about the *bZIPs* in these Amaranthaceae species is limited, and genome-wide analysis of the *bZIP* family is lacking in quinoa.

**Results:**

We identified 94 *bZIPs* in quinoa (named as *CqbZIP1*-*CqbZIP94*). All the *CqbZIPs* were phylogenetically splitted into 12 distinct subfamilies. The proportion of *CqbZIPs* was different in each subfamily, and members within the same subgroup shared conserved exon-intron structures and protein motifs. Besides, 32 duplicated *CqbZIP* gene pairs were investigated, and the duplicated *CqbZIPs* had mainly undergone purifying selection pressure, which suggested that the functions of the duplicated *CqbZIPs* might not diverge much. Moreover, we identified the *bZIP* members in 3 other Amaranthaceae species, and 41, 32, and 16 orthologous gene pairs were identified between quinoa and *S. oleracea*, *B. vulgaris*, and *A. hypochondriacus*, respectively. Among them, most were a single copy being present in *S. oleracea*, *B. vulgaris*, and *A. hypochondriacus*, and two copies being present in allotetraploid quinoa. The function divergence within the *bZIP* orthologous genes might be limited. Additionally, 11 selected *CqbZIPs* had specific spatial expression patterns, and 6 of 11 *CqbZIPs* were up-regulated in response to salt stress. Among the selected *CqbZIPs*, 3 of 4 duplicated gene pairs shared similar expression patterns, suggesting that these duplicated genes might retain some essential functions during subsequent evolution.

**Conclusions:**

The present study provided the first systematic analysis for the phylogenetic classification, motif and gene structure, expansion pattern, and expression profile of the *bZIP* family in quinoa. Our results would lay an important foundation for functional and evolutionary analysis of Cq*bZIPs*, and provide promising candidate genes for further investigation in tissue specificity and their functional involvement in quinoa’s resistance to salt stress.

## Background

Quinoa (*Chenopodium quinoa* Willd.) is a halophytic pseudocereal crop that originated from the Andean region of South America [1]. It is an allotetraploid (2n = 4x = 36) with an estimated genome size of approximately 1.5 Gbp. Quinoa belongs to the Amaranthaceae family, which also includes other economically important crops such as *Spinacia oleracea* (spinach, 2n = 2x = 12), *Beta vulgaris* (sugar beet, 2n = 2x = 18), and *Amaranthus hypochondriacus* (amaranth, 2n = 2x = 32) [2]. Quinoa produces better nutritious grains than any other major cereals [3, 4], and displays high tolerance to adverse climatic and soil conditions such as drought, soil salinity, and frost, which make it a favorable candidate for agronomic expansion into marginal lands and for identification of candidate genes facilitating stress tolerance [1, 5–7]. The potential of this emerging crop was recognized by the United Nations when 2013 was declared the International Year of Quinoa [6, 7]. To expand quinoa production worldwide and accelerate the improvement of quinoa, increasing researchers have devoted into the study of quinoa, and a draft of the *C. quinoa* genome sequence was reported recently [7], which provided the foundation for accelerating the genetic improvement of the crop and enhanced global food security for a growing world population.

Transcription factors (TFs) play vital roles in almost all plant biological processes. They are key regulators of numerous signaling networks in response to plant growth and development as well as to environmental stresses through binding to promoter and/or enhancer regions of corresponding genes to activate or repress transcription of downstream target genes [8–10]. Among several TF families that present exclusively in eukaryotes, the basic leucine zipper (bZIP) family is one of the largest and most diverse families [10–12]. The bZIP TFs contain a highly conserved bZIP domain which is composed of two structural features, a highly conserved basic region and a less conserved leucine zipper. The basic region consists of 16 amino acid residues with an invariant N-× 7-R/K motif, and is responsible for DNA binding and nuclear localization specifically. The leucine zipper includes a heptad repeat of leucines or other bulky hydrophobic amino acids for specific recognition and dimerization [10–14].

In plants, there is considerable evidence showing that bZIP TFs play crucial roles in various aspects of biological processes such as embryogenesis [15], seed maturation [16, 17], and flower and vascular development [18, 19]. On the other hand, bZIP proteins also take part in the regulation of signalling and responses to abiotic/biotic stimuli, including high salinity, drought, osmotic, cold stresses, and pathogen defense [10–12, 20]. Thus, bZIP TFs are important for plants to withstand various environmental stresses, such as salt-affected soils. Soil salinization is an increasingly serious problem, causing huge economic loss in agricultural production globally. Since quinoa can grow under harsh soil conditions and show high tolerance to salt [6, 21, 22], the crop can serve as a valuable donor of salt-tolerant genes to other crops [6].

Members of the bZIP TF family have been comprehensively identified or predicted in many eukaryotic genomes [10, 20, 23–26]. However, to our knowledge, no *bZIP* genes have been identified and isolated in quinoa so far. With quinoa genome sequencing completed, a genome-wide overview of the *bZIP* family in quinoa is urgently required. In this study, putative *bZIPs* were identified in quinoa. We conducted a relatively detailed study on the phylogenetics, gene structure, protein motif, genomic location, expansion pattern, and expression profile to evaluate the molecular evolution and biological function of the *bZIP* family in quinoa.

## Results

### Genomic identification and characterization of putative *bZIPs*

A total of 94 *bZIP* genes were confirmed and identified in quinoa (Additional file 1), and we designated these genes as *CqbZIPs*, from *CqbZIP1* to *CqbZIP94*. The primary and secondary protein structures of 94 CqbZIPs were deduced from their protein sequences (Additional file 1). The protein structures were highly diverse in all the identified CqbZIPs, and the amino acid numbers of proteins varied from 92 (CqbZIP31) to 821 (CqbZIP86), with the predicted molecular weight ranging from 10.8 kDa (CqbZIP31) to 91.6 kDa (CqbZIP86). The isoelectric points ranged from 4.38 (CqbZIP81) to 10.37 (CqbZIP42). Besides, we identified 54, 48, and 49 *bZIP* genes in *S. oleracea, B. vulgaris*, and *A. hypochondriacus*, respectively, and denoted them as *SobZIPs*, *BvbZIPs*, and *AhbZIPs*, respectively (Additional file 2).

### Phylogenetic analysis

**Fig. 1:**
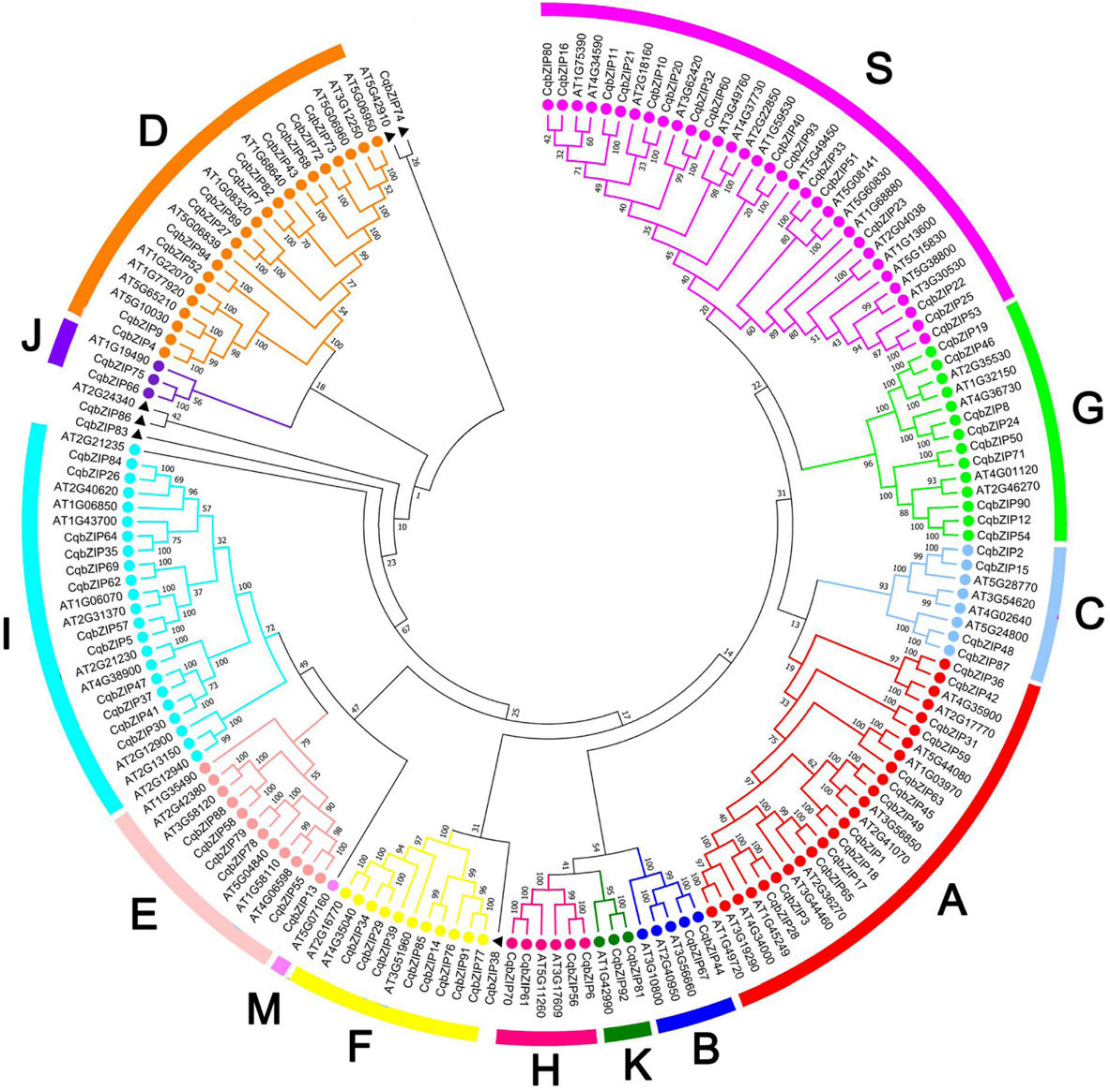
Phylogenetic relationships of the *bZIP* family in quinoa and Arabidopsis. The neighbor-joining tree was generated through the MEGA7 program based on multiple alignments with ClustalX. The subfamilies are labeled and denoted by different colors and the numbers in the clades are posterior probability values

**Fig. 2:**
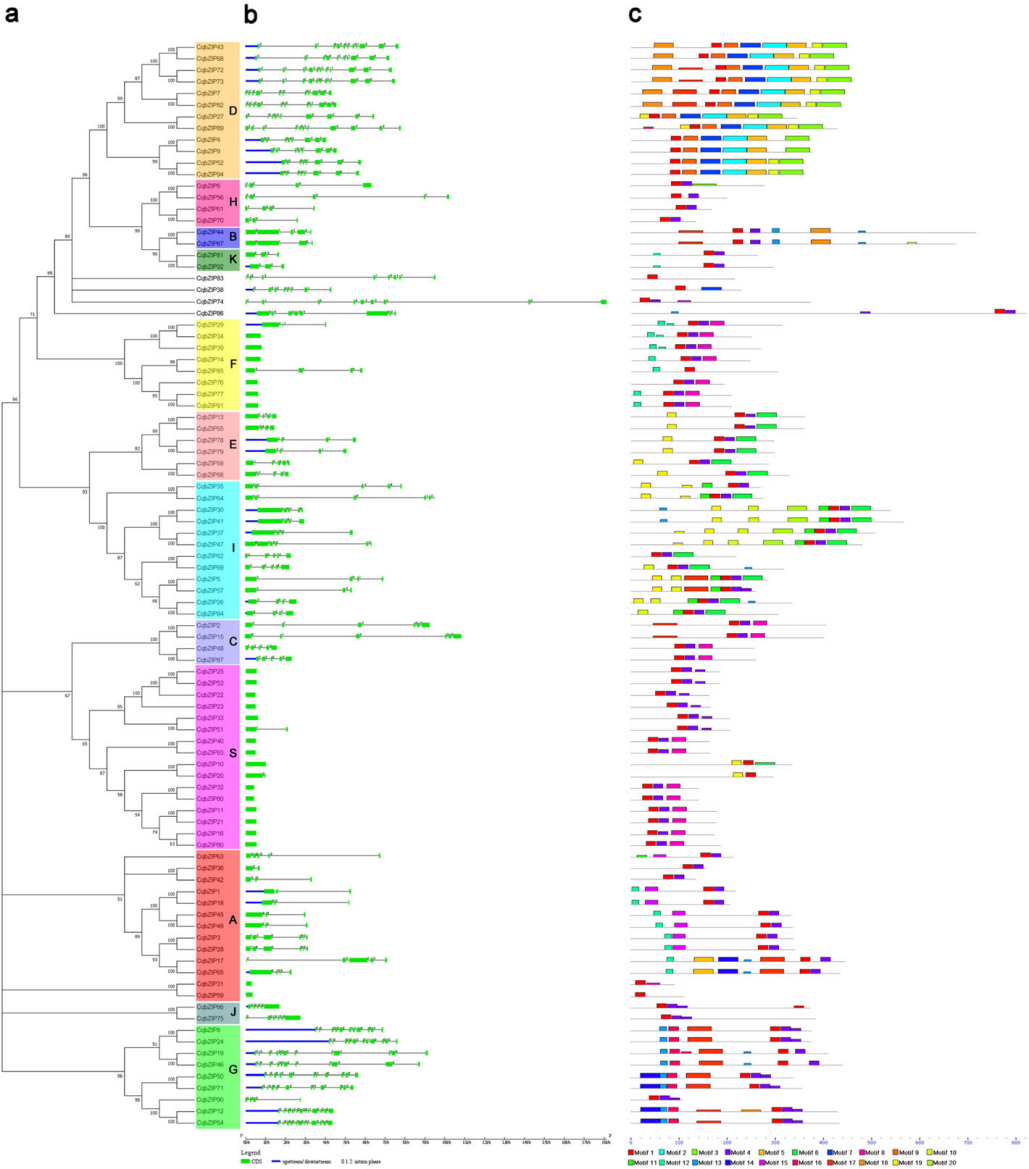
Phylogenetic relationships (**a**), gene structures (**b**), and motif compositions (**c**) of *bZIPs* in quinoa. Gene structure dynamics of *CqbZIPs* were predicted with the GSDS software. The exons are represented by green boxes and the introns are indicated by black lines. The conserved motifs were scanned with MEME. Different motifs are represented by various colored boxes

To determine the evolutionary relationships of *bZIPs* in quinoa, phylogenetic trees were constructed with the 94 CqbZIP proteins and the known bZIPs from Arabidopsis (Figs. 1 and 2a, Additional file 3).

According to the previous classification system [14], the *CqbZIP* family was divided into 12 subfamilies (Subfamily A to K, and S), and the member proportion was different in each subfamily (Additional file 4a). The Subfamily S (17%) had the most genes, followed by Subfamily A (14%), Subfamily D (13%), and Subfamily I (13%). Subfamily B (2%), Subfamily J (2%), and Subfamily K (2%) contained the least members. Besides, the *bZIPs* in spinach, sugar beet, and amaranth were phylogenetically classified (Additional file 5), and a similar member distribution in each subfamily was found in each plant (Additional file 4b-d).

### Gene structures and protein motifs of *CqbZIPs*

Gene structure and intron phase were investigated in the *CqbZIP* family (Fig. 2b). Result indicated that most of *CqbZIPs* (72 of 94 *CqbZIPs*) had introns, and the numbers of introns varied from 1 to 11. Subfamily A, B, C, E, F, H, I, J, K, and S contained 0–5 introns, whereas Subfamily D and G had 7–11 introns, except for *CqbZIP90*. Generally, most of *CqbZIP* genes in the same subgroups showed a similar exon-intron structure, and the intron patterns, formed by relative position and phase, were highly conserved within each phylogenetic subgroup.

In total, 20 conserved motifs, including the bZIP domain, were identified in the CqbZIP proteins and their multilevel consensus amino acid sequences of motifs are listed in Additional file 6. The motif distribution corresponding to the phylogenetic tree of *CqbZIP* gene family is displayed in Fig. 2c. All the CqbZIPs had Motif 1, which represented the basic region and the hinge of the bZIP domain, whilst Motif 4 and 9 corresponded to the variable motifs in the leucine zipper region across the bZIP family. For example, motif 9 only appeared in Subfamily D, while motif 4 almost appeared in the other subgroups. Moreover, some subfamily-specific motifs were identified. For instance, Motif 16 were only present in Subfamily G, Motif 15 only existed in Subfamily A, Motif 11 and 20 were only present in Subfamily I, and Motif 2, 7, and 9 only existed in Subfamily D.

### Genomic locations and gene duplications of *CqbZIPs*

**Fig. 3:**
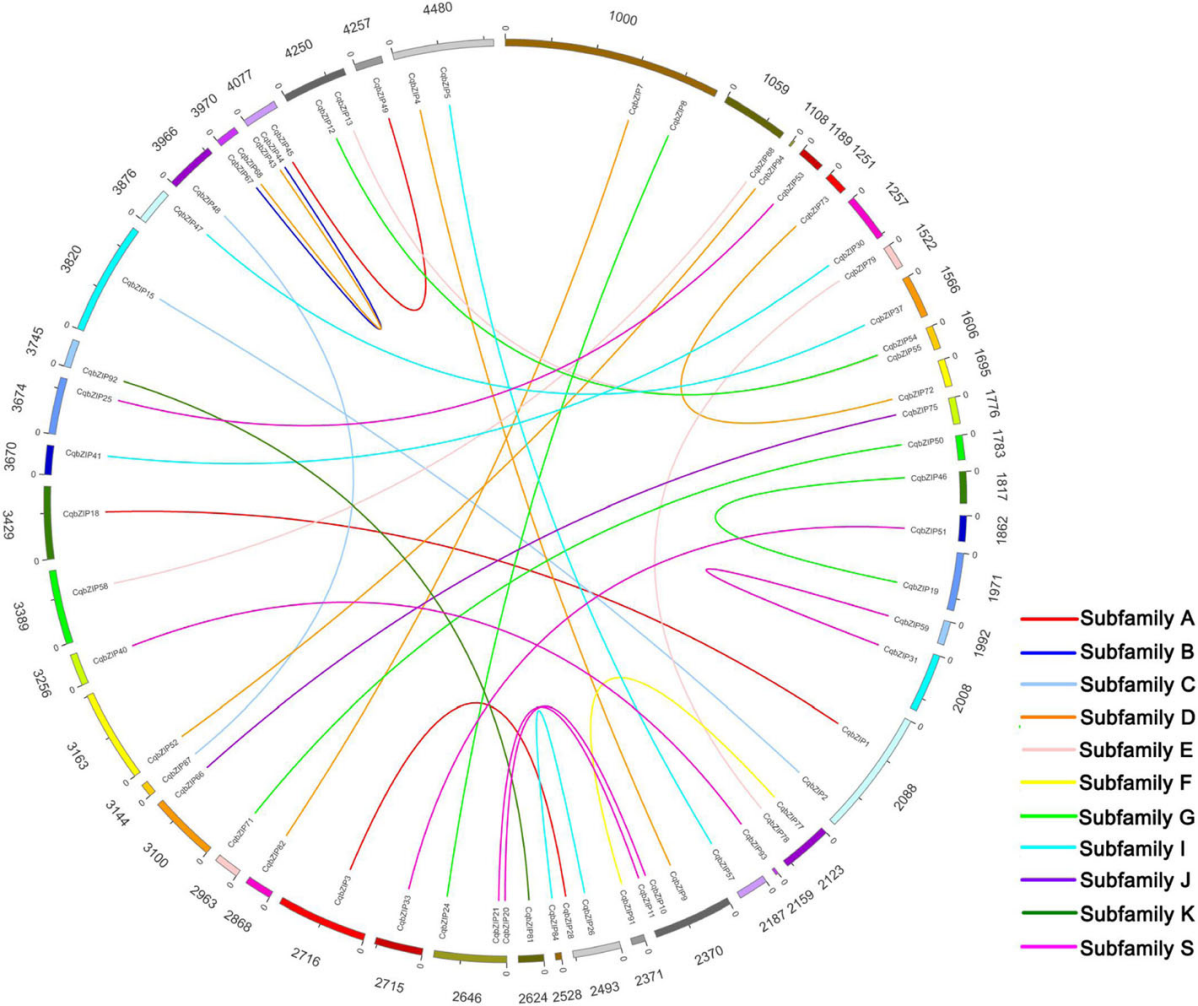
Circos diagram of duplicated *bZIP* gene pairs in quinoa. The numbers displayed outside the track of the plot indicated the scaffold numbers of quinoa. The duplicated gene pairs are joined by lines. The differently colored lines represent the subfamilies within the *CqbZIP* family

**Fig. 4:**
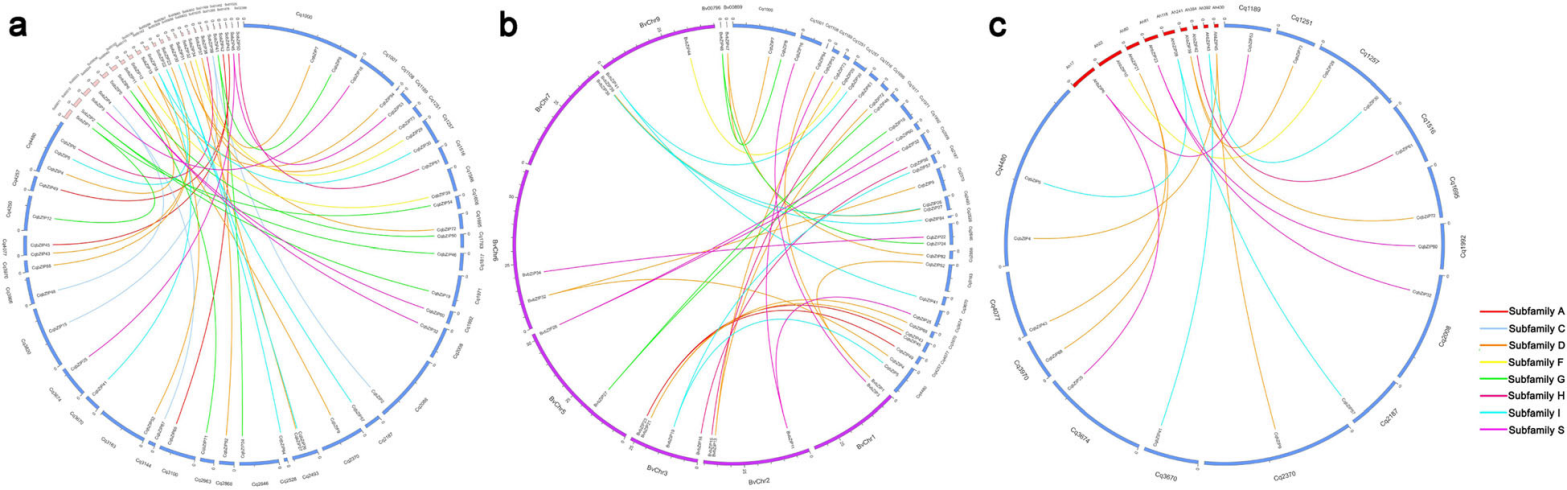
Distribution of *bZIP* orthologous gene pairs between quinoa and spinach (**a**), sugar beet (**b**), and amaranth (**c**). Lines of different colors represent subfamilies within the *bZIP* family

The genomic locations of 94 *CqbZIPs* were displayed in Additional file 7. Besides, to illustrate the expansion patterns of *CqbZIPs*, gene duplication events were investigated in the present study. As shown in Fig. 3, 32 duplicated *CqbZIP* gene pairs were identified, and the duplication events were concentrated in S, D, A, G, and I subgroups. In addition, the Ka/Ks ratios calculated for all the 32 duplicated *CqbZIP* gene pairs were less than 1 (Table 1). Moreover, orthologous relationships of *bZIPs* between quinoa and 3 other Amaranthaceae plants were analyzed, 41, 32, and 16 orthologous gene pairs were identified between quinoa and spinach, sugar beet, and amaranth, respectively (Fig. 4, Additional file 8). Among them, 17 *SobZIPs*, 13 *BvbZIPs*, and 7 *AhbZIPs* had 2 *bZIP* orthologs in quinoa. Of the orthologous gene pairs, most were distributed in Subfamily D, S, and I. All the Ka/Ks ratios except for that of *CqbZIP72*/*BvbZIP13* and *CqbZIP73*/*BvbZIP13* were less than 1.

**Table 1:** Ka/Ks analysis for duplicated gene pairs of *bZIPs* in quinoa

Duplicated gene 1	Duplicated gene 2	Subfamily	Ka	Ks	Ka/Ks	Purifing selection
CqbZIP1	CqbZIP18	A	0.0230	0.1145	0.2009	Yes
CqbZIP2	CqbZIP15	C	0.0121	0.0698	0.1734	Yes
CqbZIP3	CqbZIP28	A	0.0064	0.1116	0.0573	Yes
CqbZIP4	CqbZIP9	D	0.0035	0.1050	0.0333	Yes
CqbZIP5	CqbZIP57	I	0.0084	0.0754	0.1114	Yes
CqbZIP7	CqbZIP82	D	0.0141	0.0993	0.1420	Yes
CqbZIP8	CqbZIP24	G	0.0141	0.0641	0.2200	Yes
CqbZIP10	CqbZIP20	S	0.0649	0.1238	0.5242	Yes
CqbZIP11	CqbZIP21	S	0.0241	0.1019	0.2365	Yes
CqbZIP12	CqbZIP54	G	0.0244	0.1018	0.2397	Yes
CqbZIP13	CqbZIP55	E	0.0382	0.1099	0.3476	Yes
CqbZIP19	CqbZIP46	G	0.0096	0.0538	0.1784	Yes
CqbZIP25	CqbZIP53	S	0.0093	0.1369	0.0679	Yes
CqbZIP26	CqbZIP84	I	0.0128	0.0911	0.1405	Yes
CqbZIP30	CqbZIP41	I	0.0098	0.1256	0.0780	Yes
CqbZIP31	CqbZIP59	A	0.0571	0.0874	0.6533	Yes
CqbZIP32	CqbZIP60	S	0.0091	0.1326	0.0686	Yes
CqbZIP33	CqbZIP51	S	0.0588	0.2194	0.2680	Yes
CqbZIP37	CqbZIP47	I	0.0243	0.1185	0.2051	Yes
CqbZIP40	CqbZIP93	S	0.0519	0.0789	0.6578	Yes
CqbZIP43	CqbZIP68	D	0.0072	0.0592	0.1216	Yes
CqbZIP44	CqbZIP67	B	0.0166	0.1091	0.1522	Yes
CqbZIP45	CqbZIP49	A	0.0026	0.0820	0.0317	Yes
CqbZIP48	CqbZIP87	C	0.0273	0.1390	0.1964	Yes
CqbZIP50	CqbZIP71	G	0.0182	0.0954	0.1908	Yes
CqbZIP52	CqbZIP94	D	0.0145	0.0760	0.1908	Yes
CqbZIP58	CqbZIP88	E	0.0185	0.0949	0.1949	Yes
CqbZIP66	CqbZIP75	J	0.0330	0.1386	0.2381	Yes
CqbZIP72	CqbZIP73	D	0.0096	0.0891	0.1077	Yes
CqbZIP77	CqbZIP91	F	0.0264	0.0750	0.3520	Yes
CqbZIP78	CqbZIP79	E	0.0268	0.0398	0.6734	Yes
CqbZIP81	CqbZIP92	K	0.0000	0.0000	0.0000	Yes

### Expression patterns of *CqbZIPs*

**Fig. 5:**
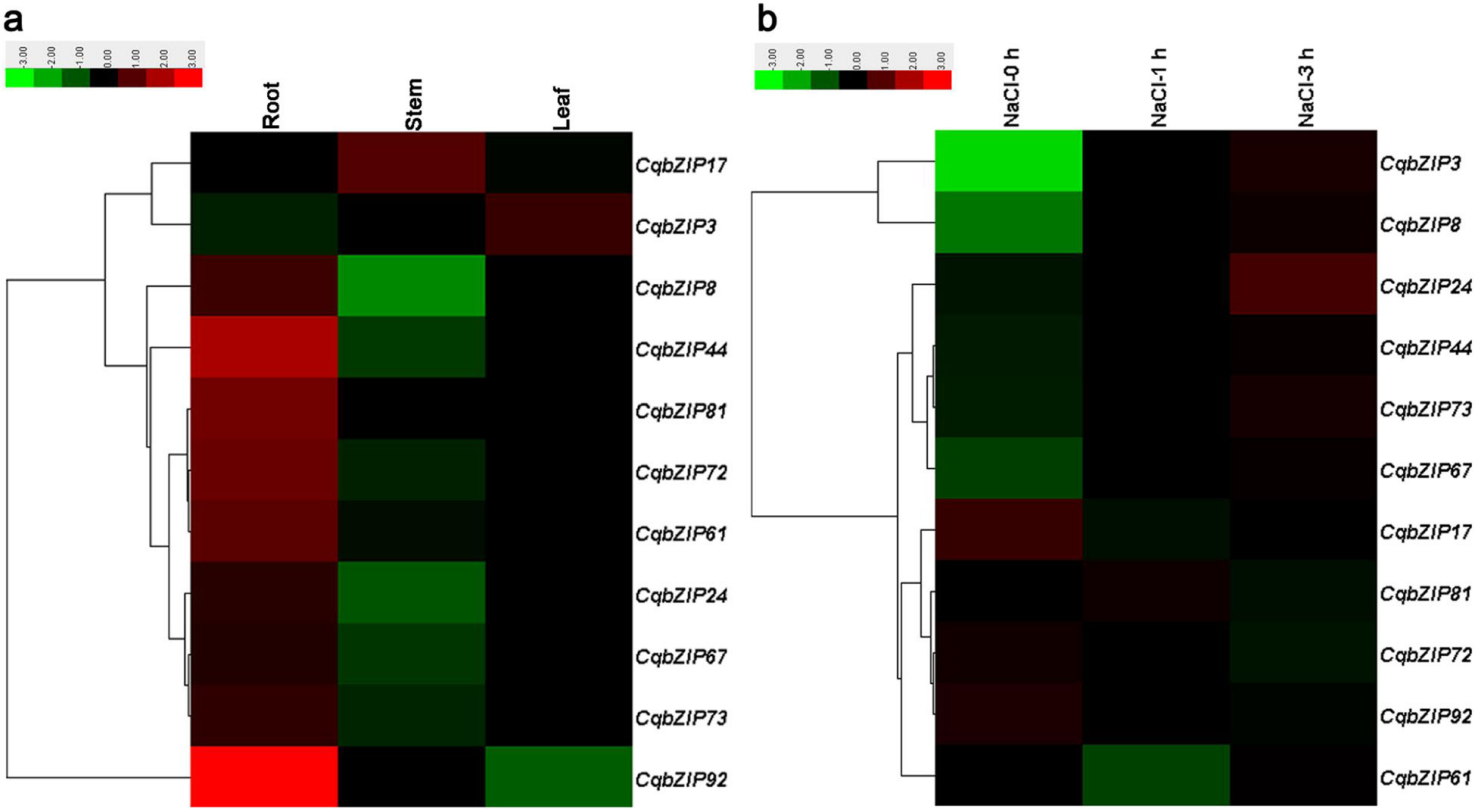
Heat map representation and hierarchical clustering of *CqbZIPs* across different tissues (**a**) and in roots under salt stress (**b**). The color bar represents the relative signal intensity value

Previous studies have reported that some *bZIP* genes such as *bZIP17* [27, 28], *bZIP49* [14], *bZIP28* [29], *bZIP60* [28, 30], *ABF1–4* [14, 31, 32], *GBF1* [33], *TGAs* [34–36], *ABI5* [37], and *HY5* [38] played a role in plant responses to salt stress as well as other abiotic stresses. In the current study, we investigated the expression patterns of 11 selected *CqbZIPs* (Fig. 5), which showed high orthology to the *bZIPs* in Arabidopsis (Fig. 1, Additional file 9). The result demonstrated that these genes showed tissue-specific expression profiles (Fig. 5a). *CqbZIP3* was mainly expressed in leaves, while *CqbZIP17* exhibited relatively high transcript abundance in young stems. Other genes such as *CqbZIP92*, *CqbZIP44*, *CqbZIP81*, *CqbZIP72*, and *CqbZIP61* were predominantly expressed in roots. Besides, the expression patterns of 11 *CqbZIPs* in roots of seedlings under salt treatment were investigated (Fig. 5b). The result showed that the expressions of the 11 *CqbZIPs* were induced or repressed after salt stress. As displayed in Fig. 5b, 6 of 11 *CqbZIPs* (*CqbZIP3*, *CqbZIP8*, *CqbZIP24*, *CqbZIP67*, *CqbZIP44*, and *CqbZIP73*) were positively responsive to salt stress, while other genes such as *CqbZIP17*, *CqbZIP72*, *CqbZIP92*, and *CqbZIP61* showed a decline in expression levels after salt stress. Moreover, the expression profiles of 4 duplicated *CqbZIP* gene pairs were compared (Additional file 10). Among them, 3 paired genes (*CqbZIP44*/*CqbZIP67*, *CqbZIP8*/*CqbZIP24*, and *CqbZIP81*/*CqbZIP92*) shared similar expression patterns (Additional file 10a-c and e-g), while this was not the case for *CqbZIP72*/*CqbZIP73*. The duplicated gene pair displayed reverse expression pattern in response to salt stress (Additional file 10h), and this might be caused by variation in gene regulation.

## Discussion

Quinoa genome is the result of genome fusion between two different doploid parent species of *Chenopodium* (*C. pallidicaule* and *C. suecicum*), each contributing to about half of the genome size [7]. In this study, a complete set of 94 *bZIP* genes were identified in quinoa, and the size of the genes is similar with that of Arabidopsis (78) [14] and rice (89) [23], but significantly lower that of soybean (160) [39] in which recent whole genome duplication (WGD) events have occurred due to palaeopolyploid, suggesting that besides the genome fusion event that happened around 4.3 million years ago, no other lineage-specific recent WGD were involved in quinoa genome evolution [40]. Besides, the encoded proteins of *CqbZIPs* showed significant differences in physical and chemical properties (Additional file 1), which were comparable with *bZIPs* genes from other plant species [23–25].

The *bZIP* members were also identified in 3 other Amaranthaceae species, and the number of *bZIPs* in allotetraploid quinoa is almost one-fold higher than that in doploid *S. oleracea* (54), *B. vulgaris* (48), and *A. hypochondriacus* (49) (Additional file 2). Among them, 12 *bZIP* subfamilies were clustered through phylogenetic analysis (Figs. 1 and 2a, Additional files 2, 3, and 5), and a similar member distribution in each subfamily was found in the 4 Amaranthaceae plants (Additional file 4). Subfamily S contained the most genes, whereas Subfamily B, J, and K had the least *bZIPs*. However, not all the subgroups were present in each plant. Compared with the members in Arabidopsis, no Subfamily M *bZIPs* existed in the 4 Amaranthaceae plants, and no Subfamily J *bZIPs* existed in *A. hypochondriacus*, suggesting that the evolution of plants not only involves gene retentions, but also is accompanied by gene losses and mutations [41].

The intron-exon pattern carries the imprint of the evolution of a gene family [42–45]. In this study, the number of introns of *CqbZIPs* varied from 0 to 11 (Fig. 2b, Additional file 3). Most of *CqbZIPs* (72 of 94 *CqbZIPs*) contained introns, only 22 of total *CqbZIP* genes were intronless. Diverse status of exon and intron splicing might be meaningful for *CqbZIP* gene evolution. Besides, the results showed that exon/intron structures of *CqbZIPs* were highly conserved within each subgroup, the genes clustered together generally possessed a similar distribution of intronic regions amid the exonic sequences. Morever, Subfamily D and G contained significantly more introns than other subfamilies, and no introns existed in most of subfamily S (14 of 16 members) and subfamily F (6 of 8 members) *CqbZIPs*, which showed a similar gene structure diversity of *bZIP*s in other species, such as cassava [20] and six legumes [26].

In this study, 20 distinct conserved motifs were also identified and classified based on sequence similarity of conserved motifs (Fig. 2c). The results indicated that all the CqbZIPs contained typical bZIP domain (Motif 1), and each subfamily had some common motifs while some subfamilies also contained the special motifs. The bZIP domain is the core of the bZIP family, which preferentially binds to the promoter of their downstream target genes on a specific *cis*- element (e.g. ABREs). The different motif compositions might contribute to the functional diversity of CqbZIP members [25]. Generally, the gene structures and motif distributions were highly conserved within each phylogenetic group, which supports their close evolutionary relationship and the classification of subfamilies.

It has been recognized that gene duplication plays an important role in the genesis of evolutionary novelty and complexity [46, 47]. In this study, gene duplication events were investigated to elucidate the expanded mechanism of the *bZIP* gene family in quinoa (Fig. 3, Table 1). We identified 32 duplicated *CqbZIP* gene pairs (Fig. 3), and the Ka/Ks ratios for all the duplicated *CqbZIP* gene pairs were less than 1 (Table 1), indicating that the *CqbZIPs* have mainly experienced purifying selection pressure with limited function divergence [41, 48, 49]. Meanwhile, the transcript levels of some duplicated *CqbZIPs* were also similar in different tissues and roots after salt stress (Additional file 10), which might be related to their highly similar protein architecture and *cis*-regulatory elements, and the result suggested that these duplicated genes might retain some essential functions during subsequent evolution [50–52].

In the Amaranthaceae family, the genera *Chenopodium* and *Spinacia* belong to Chenopoideae, the genus *Beta* belongs to Betoideae, and the genus *Amaranthus* belongs to Amaranthoideae [2]. In this study, 41, 32, and 16 *CqbZIPs* had orthologs in spinach, sugar beet, and amaranth, respectively (Fig. 4, Additional file 8), taking the evolutionary tree (Additional file 11) constructed into consideration, quinoa and spinach *bZIPs* were phylogenetically closely related compared with sugar beet and amaranth *bZIPs*, which was in line with expectations [2]. Besides, among the *bZIP* orthologous genes, most were a single copy being present in doploid spinach, sugar beet, and amaranth, and two copies being present in allotetraploid quinoa (Fig. 4, Additional file 8). The Ka/Ks ratios calculated suggested limited function divergence within the *bZIP* orthologous genes identified in this study.

As for multigene families, gene expression analysis often provides useful clues for function prediction. The result demonstrated that most of the 11 selected *CqbZIPs* had specific spatial expression patterns (Fig. 5a), which indicated their important roles in performing diverse developmental and physiological functions in quinoa. Besides, quinoa has been studied as a model to understand salt tolerance in plants, and *bZIP* genes identified in various plant species have been proven to play crucial roles in salt stress response [53–56]. In the current study, 6 of 11 *CqbZIPs*, *CqbZIP3* (orthologous to *ABF1–4*), *CqbZIP8* (orthologous to *GBF1*), *CqbZIP24* (orthologous to *GBF1*), *CqbZIP67* (orthologous to *bZIP17*, *bZIP49*, and *bZIP28*), *CqbZIP44* (orthologous to *bZIP17*, *bZIP49*, and *bZIP28*), and *CqbZIP73* (orthologous to *TGAs*) were positively regulated in response to salt stress (Fig. 5b). Our results provided evidence for selecting candidate genes for further characterization in their functional involvement in plant resistance to salt stress. On the contrary, some *CqbZIPs* were negatively responsive to salt stress, suggesting that they might be in response to other stresses or participate in other biological processes.

## Conclusions

In this report, a total of 94 *bZIPs* were isolated in quinoa. Comprehensive study of the *CqbZIPs* provided some important features of the gene family such as phylogenetic classification, expansion pattern, and expression profile. The findings of the present study could broaden our understanding on the molecular evolution and function of the *bZIP* family in quinoa, and offer a good opportunity to further investigate the *bZIP* family in plants.

## Methods

### Genomic identification of bZIP transcription factors

The quinoa (*Chenopodium quinoa* v1.0) and amaranth (*Amaranthus hypochondriacus* v1.0) genome databases were obtained from the Phytozome v12 (https://phytozome.jgi.doe.gov/pz/portal.html). The spinach (accession number: PRJNA325593) and sugar beet (accession number: PRJNA268352) genome databases were downloaded from National Center for Biotechnology Information (NCBI) (http://www.ncbi.nlm.nih.gov). The Arabidopsis bZIP sequences [14] were collected from the Arabidopsis Information Resource (TAIR) (http://www.arabidopsis.org) and were used as queries by searching against the quinoa, spinach, sugar beet, and amaranth genome databases using the BLASTP program with default parameters [57]. Afterward, the bZIP domains were confirmed by the Conserved Domain Database (CDD) program (https://www.ncbi.nlm.nih.gov/cdd) and Simple Modular Architecture Research Tool (SMART) (http://smart.embl-heidelberg.de). Finally, the confident genes were gathered and assigned as *bZIP* genes for the following analysis. Protein structures of bZIPs in quinoa were predicted with ProtParam (http://web.expasy.org/protparam/) and SOPMA (https://npsa-prabi.ibcp.fr/cgi-bin/npsa_automat.pl?page=/NPSA/npsa_sopma.html) tool.

### Phylogenetic classification and structural analysis

All the bZIP sequences identified in this study were aligned using ClustalX version 2.1 [58]. Then, neighbor-joining phylogenetic trees were constructed by MEGA7 (Molecular Evolutionary Genetics Analysis) [59]. Bootstrap analysis was conducted with 1000 replicates to assess the statistical support for each node. The conserved motifs of the bZIP proteins in quinoa were scanned using the online Multiple Em for Motif Elicitation (MEME) program (http://meme-suite.org/tools/meme), parameters were set based on a previous study [41]. To illustrate exon-intron organization for quinoa *bZIPs*, the Gene Structure Display Server (GSDS) tool (http://gsds.cbi.pku.edu.cn/) was employed by comparing the predicted coding sequences with their corresponding genomic sequences.

### Chromosomal mapping and gene duplications

Specific chromosomal positions of the quinoa and amaranth *bZIPs* were downloaded from the Phytozome database, and the chromosome location information of *bZIPs* in spinach and sugar beet were searched in NCBI. Duplicated gene pairs were searched via BLASTP and phylogenetic analysis [49], and illustrated with the Circos program [60]. The evolutionary rates, Ka (non-synonymous substitution rate) and Ks (synonymous substitution rate) were estimated by DnaSP v5.0 software [61], and the Ka/Ks ratio was calculated to assess the selection pressure for each duplicated gene pair.

### Plant materials, RNA extraction, and quantitative real-time PCR

The white quinoa seeds (ymsBLM-2) were kindly supplied by Maize Research Institute, Shanxi Academy of Agricultural Sciences. Sterilized seeds were cultivated in a growth chamber at controlled conditions (24 °C day/22 °C night, 16 h light/8 h dark). RNA samples were collected from 4 to 5-leaf-stage seedlings. Roots, stems, leaves, and the roots exposed to 300 mM NaCl (salt stress) for 0 h, 1 h, and 3 h were harvested. Afterward, the total RNAs were extracted using an RNeasy Plant Mini Kit (QIAGEN), and preparation of cDNA was performed using SuperScript™ III Reverse Transcriptase kit (Invitrogen). Gene-specific primers were designed (Additional file 12) and then synthesized commercially (HUADA Gene, Beijing, China). Quantitative real-time PCR (qRT-PCR) was performed with 2× QuantiTect SYBR Green PCR mix (QIAGEN) and ABI ViiA 7 Real-time PCR system (Applied Biosystems, USA) by strictly following the manufacturer’s instructions. The qRT-PCR machine was set with 40 cycles and an annealing temperature of 60 °C. Relative gene transcript levels were measured as 2^−⊿⊿Ct^ [62], and normalized against Elongation Factor 1 alpha (*EF1α*) gene transcript levels. Each experiment was repeated in triplicate using independent RNA samples. The expression patterns of the *bZIPs* in quinoa were clustered using the Cluster 3.0 software [63].

## Supplementary information

**Additional file 1.** The structural analysis of bZIPs identified in this study.

**Additional file 2. **The *bZIPs* identified in spinach, sugar beet, and amaranth.

**Additional file 3. **The classification and gene structures of *bZIPs* in quinoa.

**Additional file 4. **The percentage of members in each *bZIP* subfamily in quinoa, spinach, sugar beet, and amaranth.

**Additional file 5.** Phylogenetic relationships of the bZIPs in spinach, sugar beet, amaranth, and Arabidopsis.

**Additional file 6.** Multilevel consensus sequence and their logo of conserved motifs identified in CqbZIP proteins as predicted by MEME program.

**Additional file 7. **Genomic locations of *bZIPs* in quinoa.

**Additional file 8. **Ka/Ks analysis for orthologous *bZIP* gene pairs between quinoa and spinach, sugar beet, and amaranth.

**Additional file 9.** Orthology information of CqbZIPs and AtbZIPs from BLASTP.

**Additional file 10. **Expression patterns of some duplicated *CqbZIP* genes in different organs and in roots under salt stress treatment.

**Additional file 11.** Phylogenetic relationships of the bZIPs in quinoa, spinach, sugar beet, and amaranth.

**Additional file 12.** PCR primers used for qRT-PCR in this study.

## Data Availability

The Arabidopsis bZIP protein sequences were collected from the Arabidopsis information source (TAIR) database (http://www.arabidopsis.org). The genome sequences of quinoa (*Chenopodium quinoa* v1.0) and amaranth (*Amaranthus hypochondriacus* v1.0) were downloaded from Phytozome v12 (https://phytozome.jgi.doe.gov/pz/portal.html). The genome sequences of spinach (accession number: PRJNA325593) and sugar beet (accession number: PRJNA268352) were downloaded from National Center for Biotechnology Information (NCBI) (http://www.ncbi.nlm.nih.gov). All data used during the current study are included in this published article and its additional files or are available from the corresponding author on reasonable request.

## Abbreviations

bZIP: Basic leucine zipper
TF: Transcription factor
NCBI: National Center for Biotechnology Information
TAIR: The Arabidopsis Information Resource
CDD: Conserved Domain Database
SMART: Simple Modular Architecture Research Tool
MEGA: Molecular Evolutionary Genetics Analysis
MEME: Multiple Em for Motif Elicitation
GSDS: Gene Structure Display Server
Ka/Ks: Non-synonymous substitution rate/synonymous substitution rate
*EF1α*: Elongation Factor 1 alpha gene
qRT-PCR: Quantitative real-time PCR
