# Supplementary material for: Genome-wide survey, characterization, and expression analysis of bZIP transcription factors in Chenopodium quinoa
Source: BMC Plant Biol. 2020 Sep 1;20:405. doi: 10.1186/s12870-020-02620-z (PMC7466520; doi:10.1186/s12870-020-02620-z)
Supplement: Supplementary file 1 — Additional file 1. The structural analysis of bZIPs identified in this study. [file 12870_2020_2620_MOESM1_ESM.doc]

**Additional file 1:** The structural analysis of bZIPs identified in this study

| Name | Locus name | Protein length (AA) | α-helix | Extended strand | β-turn | Random coil | Molecular weight | Theoretical pI |
| --- | --- | --- | --- | --- | --- | --- | --- | --- |
| CqbZIP1 | AUR62000445 | 219 | 45.21% | 3.65% | 1.83% | 49.32% | 24761.10 | 8.62 |
| CqbZIP2 | AUR62001010 | 406 | 35.22% | 0.74% | 1.48% | 62.56% | 43222.01 | 5.73 |
| CqbZIP3 | AUR62001422 | 338 | 35.50% | 7.69% | 2.37% | 54.44% | 35878.19 | 9.23 |
| CqbZIP4 | AUR62002046 | 374 | 63.64% | 5.08% | 1.87% | 29.41% | 42705.54 | 6.48 |
| CqbZIP5 | AUR62002345 | 285 | 46.32% | 3.86% | 3.51% | 46.32% | 30671.11 | 5.16 |
| CqbZIP6 | AUR62002522 | 278 | 36.33% | 15.83% | 5.40% | 42.45% | 31212.06 | 5.25 |
| CqbZIP7 | AUR62002902 | 446 | 53.14% | 10.54% | 2.47% | 33.86% | 49821.00 | 6.24 |
| CqbZIP8 | AUR62003183 | 377 | 29.44% | 7.16% | 2.65% | 60.74% | 40244.24 | 5.20 |
| CqbZIP9 | AUR62003749 | 376 | 60.90% | 5.85% | 1.33% | 31.91% | 42984.82 | 6.73 |
| CqbZIP10 | AUR62004028 | 336 | 29.17% | 14.58% | 2.68% | 53.57% | 38019.74 | 7.68 |
| CqbZIP11 | AUR62004100 | 180 | 48.89% | 12.78% | 5.00% | 33.33% | 20197.16 | 5.02 |
| CqbZIP12 | AUR62004443 | 431 | 29.00% | 5.34% | 2.09% | 63.57% | 45761.88 | 6.64 |
| CqbZIP13 | AUR62004622 | 361 | 37.40% | 2.22% | 1.11% | 59.28% | 39856.61 | 6.76 |
| CqbZIP14 | AUR62004732 | 247 | 33.60% | 6.88% | 2.83% | 56.68% | 28444.74 | 5.91 |
| CqbZIP15 | AUR62004948 | 401 | 41.40% | 2.00% | 1.25% | 55.36% | 42790.39 | 5.73 |
| CqbZIP16 | AUR62005989 | 175 | 60.57% | 5.71% | 2.29% | 31.43% | 19796.02 | 6.13 |
| CqbZIP17 | AUR62006172 | 446 | 38.79% | 5.83% | 1.57% | 53.81% | 48793.14 | 7.20 |
| CqbZIP18 | AUR62006793 | 208 | 49.52% | 1.92% | 1.92% | 46.63% | 23368.58 | 6.78 |
| CqbZIP19 | AUR62007361 | 412 | 18.69% | 6.31% | 0.00% | 75.00% | 43902.77 | 5.69 |
| CqbZIP20 | AUR62007536 | 296 | 20.95% | 14.19% | 3.38% | 61.49% | 33046.71 | 6.06 |
| CqbZIP21 | AUR62007613 | 179 | 48.60% | 13.41% | 3.35% | 34.64% | 20026.90 | 5.16 |
| CqbZIP22 | AUR62007834 | 163 | 62.58% | 3.07% | 1.84% | 32.52% | 18802.85 | 6.21 |
| CqbZIP23 | AUR62007835 | 166 | 68.67% | 0.00% | 0.00% | 31.33% | 19709.65 | 5.64 |
| CqbZIP24 | AUR62008004 | 376 | 29.79% | 6.65% | 1.86% | 61.70% | 39909.96 | 5.28 |
| CqbZIP25 | AUR62009445 | 184 | 60.87% | 2.72% | 0.00% | 36.41% | 21445.68 | 5.13 |
| CqbZIP26 | AUR62009733 | 337 | 36.80% | 2.97% | 2.37% | 57.86% | 37276.27 | 5.89 |
| CqbZIP27 | AUR62009764 | 346 | 64.74% | 5.20% | 2.31% | 27.75% | 38935.11 | 8.94 |
| CqbZIP28 | AUR62009948 | 341 | 37.24% | 8.21% | 2.64% | 51.91% | 36238.62 | 9.36 |
| CqbZIP29 | AUR62010042 | 315 | 28.25% | 14.92% | 3.49% | 53.33% | 34196.09 | 6.07 |
| CqbZIP30 | AUR62010368 | 541 | 29.94% | 3.51% | 1.11% | 65.43% | 58501.39 | 6.26 |
| CqbZIP31 | AUR62011804 | 92 | 91.30% | 2.17% | 0.00% | 6.52% | 10825.26 | 9.30 |
| CqbZIP32 | AUR62011806 | 141 | 65.96% | 0.00% | 1.42% | 32.62% | 16321.51 | 6.12 |
| CqbZIP33 | AUR62012705 | 206 | 47.09% | 9.22% | 2.43% | 41.26% | 23617.39 | 5.57 |
| CqbZIP34 | AUR62013034 | 249 | 31.33% | 11.24% | 1.61% | 55.82% | 27322.47 | 5.73 |
| CqbZIP35 | AUR62013553 | 269 | 39.78% | 8.92% | 2.23% | 49.07% | ** | ** |
| CqbZIP36 | AUR62013917 | 158 | 49.37% | 11.39% | 0.00% | 39.24% | 18116.22 | 6.85 |
| CqbZIP37 | AUR62014406 | 509 | 34.77% | 3.54% | 2.36% | 59.33% | 55252.11 | 6.45 |
| CqbZIP38 | AUR62014528 | 229 | 62.01% | 14.41% | 4.80% | 18.78% | 25733.15 | 9.72 |
| CqbZIP39 | AUR62014567 | 270 | 31.48% | 10.37% | 2.96% | 55.19% | 30189.47 | 6.00 |
| CqbZIP40 | AUR62016126 | 164 | 62.20% | 6.10% | 3.05% | 28.66% | 18315.67 | 9.28 |
| CqbZIP41 | AUR62016851 | 568 | 30.99% | 3.70% | 1.06% | 64.26% | ** | ** |
| CqbZIP42 | AUR62018275 | 135 | 50.37% | 8.15% | 0.74% | 40.74% | 15326.46 | 10.37 |
| CqbZIP43 | AUR62018350 | 450 | 55.78% | 6.44% | 2.22% | 35.56% | 49782.65 | 6.78 |
| CqbZIP44 | AUR62018385 | 718 | 19.92% | 13.93% | 3.20% | 62.95% | 77679.81 | 5.61 |
| CqbZIP45 | AUR62018468 | 334 | 31.74% | 3.59% | 1.80% | 62.87% | 36579.86 | 6.99 |
| CqbZIP46 | AUR62018627 | 441 | 22.22% | 7.94% | 1.59% | 68.25% | 47377.72 | 5.83 |
| CqbZIP47 | AUR62019143 | 481 | 34.93% | 3.95% | 2.08% | 59.04% | 51628.75 | 6.30 |
| CqbZIP48 | AUR62020638 | 256 | 43.36% | 10.55% | 4.69% | 41.41% | 27777.49 | 5.19 |
| CqbZIP49 | AUR62020955 | 339 | 30.97% | 7.08% | 2.065 | 59.88% | 37059.39 | 6.99 |
| CqbZIP50 | AUR62021054 | 338 | 31.07% | 2.96% | 1.48% | 64.50% | 36301.94 | 5.41 |
| CqbZIP51 | AUR62021950 | 208 | 53.37% | 7.69% | 2.88% | 36.06% | 24281.41 | 5.88 |
| CqbZIP52 | AUR62022327 | 362 | 63.26% | 6.35% | 2.21% | 28.18% | 40957.37 | 5.82 |
| CqbZIP53 | AUR62023129 | 184 | 57.61% | 2.72% | 0.00% | 39.67% | 21404.67 | 5.13 |
| CqbZIP54 | AUR62023507 | 433 | 26.56% | 3.46% | 1.62% | 68.36% | 45961.13 | 7.05 |
| CqbZIP55 | AUR62023517 | 360 | 33.33% | 3.61% | 1.67% | 61.39% | 39842.57 | 6.47 |
| CqbZIP56 | AUR62023672 | 201 | 46.27% | 10.45% | 4.48% | 38.81% | 22267.21 | 6.64 |
| CqbZIP57 | AUR62023846 | 261 | 41.38% | 3.07% | 0.77% | 54.79% | 28089.07 | 5.58 |
| CqbZIP58 | AUR62024071 | 287 | 34.84% | 6.97% | 3.14% | 55.05% | 32299.86 | 5.74 |
| CqbZIP59 | AUR62024650 | 112 | 83.04% | 6.25% | 5.36% | 5.36% | 12955.65 | 9.26 |
| CqbZIP60 | AUR62024652 | 141 | 63.83% | 0.71% | 2.84% | 32.62% | 16369.55 | 6.12 |
| CqbZIP61 | AUR62024899 | 168 | 52.98% | 0.00% | 0.00% | 47.02% | 18249.98 | 9.74 |
| CqbZIP62 | AUR62024931 | 219 | 45.21% | 8.22% | 3.20% | 43.38% | 24635.49 | 5.02 |
| CqbZIP63 | AUR62026695 | 213 | 60.56% | 6.10% | 2.35% | 30.99% | 23879.35 | 6.24 |
| CqbZIP64 | AUR62026729 | 276 | 43.12% | 6.88% | 2.17% | 47.83% | 30222.72 | 6.46 |
| CqbZIP65 | AUR62028537 | 436 | 32.57% | 3.21% | 1.38% | 62.84% | ** | ** |
| CqbZIP66 | AUR62028570 | 374 | 33.69% | 7.22% | 0.53% | 58.56% | 42168.61 | 8.93 |
| CqbZIP67 | AUR62030278 | 675 | 22.52% | 13.63% | 3.56% | 60.30% | 73115.62 | 5.51 |
| CqbZIP68 | AUR62030316 | 424 | 59.43% | 6.37% | 1.65% | 32.55% | 46899.57 | 6.48 |
| CqbZIP69 | AUR62030616 | 317 | 41.64% | 8.83% | 1.58% | 47.95% | 35977.09 | 5.48 |
| CqbZIP70 | AUR62030640 | 135 | 54.81% | 1.48% | 0.00% | 43.70% | 14901.51 | 10.13 |
| CqbZIP71 | AUR62031478 | 357 | 33.33% | 7.84% | 3.08% | 55.74% | 38203.25 | 5.67 |
| CqbZIP72 | AUR62031746 | 455 | 54.95% | 5.93% | 1.32% | 37.80% | 49859.83 | 7.15 |
| CqbZIP73 | AUR62032631 | 460 | 54.57% | 5.87% | 1.30% | 38.26% | 50531.54 | 8.46 |
| CqbZIP74 | AUR62033078 | 372 | 47.04% | 15.59% | 4.30% | 33.06% | 42022.26 | 6.07 |
| CqbZIP75 | AUR62033525 | 384 | 31.51% | 8.85% | 0.52% | 59.11% | 42912.25 | 9.09 |
| CqbZIP76 | AUR62034128 | 194 | 41.24% | 9.28% | 4.64% | 44.85% | 22204.52 | 4.97 |
| CqbZIP77 | AUR62034129 | 209 | 35.41% | 15.79% | 2.87% | 45.93% | 23879.73 | 6.26 |
| CqbZIP78 | AUR62034166 | 298 | 44.30% | 3.69% | 0.67% | 51.34% | 33519.61 | 6.46 |
| CqbZIP79 | AUR62034488 | 299 | 44.48% | 3.01% | 1.67% | 50.84% | 33583.69 | 7.71 |
| CqbZIP80 | AUR62034516 | 188 | 54.26% | 9.57% | 2.66% | 33.51% | 21252.39 | 5.83 |
| CqbZIP81 | AUR62035186 | 264 | 32.95% | 10.61% | 3.79% | 52.65% | 29380.57 | 4.38 |
| CqbZIP82 | AUR62035261 | 440 | 55.45% | 10.91% | 1.82% | 31.82% | 49079.36 | 6.71 |
| CqbZIP83 | AUR62035788 | 216 | 55.09% | 15.28% | 3.24% | 26.39% | 24682.74 | 9.47 |
| CqbZIP84 | AUR62036211 | 307 | 41.37% | 2.93% | 1.95% | 53.75% | 33778.28 | 5.61 |
| CqbZIP85 | AUR62037528 | 305 | 27.87% | 14.10% | 7.54% | 50.49% | 33857.61 | 4.78 |
| CqbZIP86 | AUR62037870 | 821 | 27.53% | 14.62% | 4.87% | 52.98% | 91612.23 | 6.29 |
| CqbZIP87 | AUR62038742 | 262 | 38.93% | 11.45% | 2.67% | 46.95% | 28374.20 | 5.08 |
| CqbZIP88 | AUR62038993 | 330 | 34.24% | 4.85% | 2.73% | 58.18% | 37468.62 | 6.78 |
| CqbZIP89 | AUR62039211 | 430 | 55.58% | 8.60% | 2.79% | 33.02% | 48173.70 | 9.23 |
| CqbZIP90 | AUR62039832 | 109 | 71.56% | 11.01% | 3.67% | 13.76% | 12150.56 | 6.62 |
| CqbZIP91 | AUR62041291 | 209 | 36.36% | 14.35% | 3.83% | 45.45% | 23887.54 | 5.52 |
| CqbZIP92 | AUR62041341 | 297 | 31.31% | 9.76% | 1.35% | 57.58% | 33311.28 | 4.58 |
| CqbZIP93 | AUR62041399 | 165 | 50.30% | 9.09% | 2.42% | 38.18% | 18411.91 | 9.49 |
| CqbZIP94 | AUR62043508 | 362 | 64.36% | 6.35% | 2.21% | 27.07% | 40961.39 | 6.05 |
