# Supplementary material for: Genome-wide survey, characterization, and expression analysis of bZIP transcription factors in Chenopodium quinoa
Source: BMC Plant Biol. 2020 Sep 1;20:405. doi: 10.1186/s12870-020-02620-z (PMC7466520; doi:10.1186/s12870-020-02620-z)
Supplement: Supplementary file 2 — Additional file 2. The bZIPs identified in spinach, sugar beet, and amaranth. [file 12870_2020_2620_MOESM2_ESM.doc]

**Additional file 2:** The *bZIPs* identified in spinach, beet, and amaranth in this study

| Name | Locus Name | Subfamily | Chromosome Name | Gene Start (bp) | Gene End (bp) |
| --- | --- | --- | --- | --- | --- |
| *SobZIP1* | LOC110790332 | G | SpoScf_00011 | 1700970 | 1705523 |
| *SobZIP2* | LOC110791421 | G | SpoScf_00012 | 382221 | 389782 |
| *SobZIP3* | LOC110797555 | S | SpoScf_00023 | 673698 | 674607 |
| *SobZIP4* | LOC110783906 | C | SpoScf_00034 | 90003 | 95381 |
| *SobZIP5* | LOC110777068 | S | SpoScf_00036 | 888175 | 889431 |
| *SobZIP6* | LOC110779939 | G | SpoScf_00040 | 605320 | 612766 |
| *SobZIP7* | LOC110784681 | I | SpoScf_00057 | 1015263 | 1027965 |
| *SobZIP8* | LOC110784772 | F | SpoScf_00060 | 786470 | 797321 |
| *SobZIP9* | LOC110784826 | A | SpoScf_00061 | 676646 | 686507 |
| *SobZIP10* | LOC110785151 | E | SpoScf_00070 | 136895 | 139750 |
| *SobZIP11* | LOC110786139 | D | SpoScf_00107 | 276256 | 282625 |
| *SobZIP12* | LOC110786814 | F | SpoScf_00112 | 123604 | 125456 |
| *SobZIP13* | LOC110787215 | A | SpoScf_00126 | 492356 | 496487 |
| *SobZIP14* | LOC110787039 | E | SpoScf_00127 | 705420 | 710229 |
| *SobZIP15* | LOC110787025 | D | SpoScf_00130 | 647119 | 659190 |
| *SobZIP16* | LOC110787588 | E | SpoScf_00146 | 293941 | 295178 |
| *SobZIP17* | LOC110794990 | K | SpoScf_00158 | 685049 | 689396 |
| *SobZIP18* | LOC110787717 | I | SpoScf_00162 | 240288 | 243334 |
| *SobZIP19* | LOC110795200 | A | SpoScf_00244 | 537216 | 537572 |
| *SobZIP20* | LOC110801746 | E | SpoScf_00247 | 243513 | 249407 |
| *SobZIP21* | LOC110789123 | A | SpoScf_00280 | 318818 | 330731 |
| *SobZIP22* | LOC110802272 | I | SpoScf_00299 | 459969 | 466574 |
| *SobZIP23* | LOC110795989 | D | SpoScf_00309 | 64970 | 71461 |
| *SobZIP24* | LOC110796393 | E | SpoScf_00325 | 313314 | 320016 |
| *SobZIP25* | LOC110802935 | I | SpoScf_00389 | 293110 | 300492 |
| *SobZIP26* | LOC110792423 | S | SpoScf_00403 | 273324 | 274685 |
| *SobZIP27* | LOC110803594 | A | SpoScf_00449 | 415797 | 418524 |
| *SobZIP28* | LOC110796934 | S | SpoScf_00507 | 176070 | 176830 |
| *SobZIP29* | LOC110796936 | S | SpoScf_00507 | 132688 | 133474 |
| *SobZIP30* | LOC110797390 | I | SpoScf_00567 | 232126 | 235033 |
| *SobZIP31* | LOC110797701 | D | SpoScf_00658 | 224590 | 235167 |
| *SobZIP32* | LOC110797839 | D | SpoScf_00663 | 231427 | 238089 |
| *SobZIP33* | LOC110804885 | A | SpoScf_00724 | 148595 | 157049 |
| *SobZIP34* | LOC110791531 | F | SpoScf_00822 | 60177 | 64815 |
| *SobZIP35* | LOC110805171 | F | SpoScf_00823 | 181675 | 183068 |
| *SobZIP36* | LOC110791600 | S | SpoScf_00853 | 164213 | 165882 |
| *SobZIP37* | LOC110805644 | D | SpoScf_00932 | 196517 | 201628 |
| *SobZIP38* | LOC110805805 | H | SpoScf_01035 | 110268 | 114051 |
| *SobZIP39* | LOC110774937 | C | SpoScf_01169 | 169959 | 182323 |
| *SobZIP40* | LOC110793072 | S | SpoScf_01315 | 83709 | 84233 |
| *SobZIP41* | LOC110775112 | G | SpoScf_01355 | 123052 | 132395 |
| *SobZIP42* | LOC110800189 | A | SpoScf_01462 | 71304 | 77337 |
| *SobZIP43* | LOC110793657 | A | SpoScf_01478 | 109630 | 113894 |
| *SobZIP44* | LOC110775440 | I | SpoScf_01513 | 71554 | 73851 |
| *SobZIP45* | LOC110800298 | S | SpoScf_01525 | 33775 | 34621 |
| *SobZIP46* | LOC110800466 | J | SpoScf_01617 | 40033 | 46014 |
| *SobZIP47* | LOC110793874 | S | SpoScf_01671 | 3313 | 3648 |
| *SobZIP48* | LOC110800711 | D | SpoScf_01795 | 14352 | 26961 |
| *SobZIP49* | LOC110794012 | A | SpoScf_01851 | 63841 | 65808 |
| *SobZIP50* | LOC110777315 | H | SpoScf_02388 | 14323 | 18221 |
| *SobZIP51* | LOC110777732 | S | SpoScf_02523 | 6477 | 8108 |
| *SobZIP52* | LOC110779820 | S | SpoScf_04504 | 5735 | 6718 |
| *SobZIP53* | LOC110780112 | B | SpoScf_05189 | 1964 | 5266 |
| *SobZIP54* | LOC110780612 | A | SpoScf_08670 | 391 | 2739 |
| *BvbZIP1* | LOC104905689 | D | Chr1 | 501404 | 512443 |
| *BvbZIP2* | LOC104896130 | E | Chr1 | 1455728 | 1459440 |
| *BvbZIP3* | LOC104888155 | S | Chr1 | 3093992 | 3095517 |
| *BvbZIP4* | LOC104886686 | G | Chr1 | 3977916 | 3982942 |
| *BvbZIP5* | LOC104887833 | I | Chr1 | 5540972 | 5546329 |
| *BvbZIP6* | LOC104889396 | S | Chr1 | 7653114 | 7654814 |
| *BvbZIP7* | LOC104895660 | I | Chr1 | 18474752 | 18486050 |
| *BvbZIP8* | LOC104895875 | A | Chr1 | 19888105 | 19896453 |
| *BvbZIP9* | LOC104899269 | K | Chr1 | 29718507 | 29722385 |
| *BvbZIP10* | LOC104905249 | F | Chr2 | 2777401 | 2779008 |
| *BvbZIP11* | LOC104907325 | S | Chr2 | 5556010 | 5557185 |
| *BvbZIP12* | LOC104886702 | C | Chr2 | 15713633 | 15722655 |
| *BvbZIP13* | LOC104887563 | D | Chr2 | 36849298 | 36858494 |
| *BvbZIP14* | LOC104887633 | I | Chr2 | 37909453 | 37913193 |
| *BvbZIP15* | LOC104887661 | H | Chr2 | 38413121 | 38417226 |
| *BvbZIP16* | LOC104906977 | H | Chr3 | 459791 | 463907 |
| *BvbZIP17* | LOC104888309 | G | Chr3 | 4903671 | 4911198 |
| *BvbZIP18* | LOC104888416 | S | Chr3 | 6266841 | 6267350 |
| *BvbZIP19* | LOC104888846 | I | Chr3 | 12517919 | 12529117 |
| *BvbZIP20* | LOC104889231 | D | Chr3 | 19135726 | 19150319 |
| *BvbZIP21* | LOC104889491 | D | Chr3 | 23089301 | 23097287 |
| *BvbZIP22* | LOC104889531 | B | Chr3 | 23723459 | 23726886 |
| *BvbZIP23* | LOC104889671 | A | Chr3 | 25013663 | 25018273 |
| *BvbZIP24* | LOC104907452 | F | Chr4 | 1102915 | 1103742 |
| *BvbZIP25* | LOC104890911 | C | Chr4 | 12442060 | 12447944 |
| *BvbZIP26* | LOC104891424 | E | Chr4 | 24868943 | 24873916 |
| *BvbZIP27* | LOC104893043 | G | Chr5 | 16232097 | 16244958 |
| *BvbZIP28* | LOC104894764 | S | Chr5 | 51617619 | 51618983 |
| *BvbZIP29* | LOC104894976 | S | Chr6 | 1714961 | 1716254 |
| *BvbZIP30* | LOC104895073 | S | Chr6 | 2809761 | 2811097 |
| *BvbZIP31* | LOC104895559 | A | Chr6 | 8140566 | 8161505 |
| *BvbZIP32* | LOC104895725 | D | Chr6 | 10485448 | 10490650 |
| *BvbZIP33* | LOC104896277 | S | Chr6 | 20681507 | 20682236 |
| *BvbZIP34* | LOC104896274 | S | Chr6 | 20809273 | 20810261 |
| *BvbZIP35* | LOC104898371 | A | Chr7 | 3838768 | 3848663 |
| *BvbZIP36* | LOC104898393 | J | Chr7 | 4346206 | 4351489 |
| *BvbZIP37* | LOC104899695 | A | Chr7 | 35447527 | 35451803 |
| *BvbZIP38* | LOC104900287 | I | Chr7 | 43158294 | 43161957 |
| *BvbZIP39* | LOC104900318 | D | Chr7 | 43480497 | 43492445 |
| *BvbZIP40* | LOC104900999 | S | Chr8 | 8997688 | 8998614 |
| *BvbZIP41* | LOC104883107 | I | Chr9 | 13148 | 16084 |
| *BvbZIP42* | LOC104903082 | A | Chr9 | 11871350 | 11882197 |
| *BvbZIP43* | LOC104903424 | E | Chr9 | 21452927 | 21456740 |
| *BvbZIP44* | LOC104903923 | F | Chr9 | 33644446 | 33649387 |
| *BvbZIP45* | LOC104904696 | E | Chr9 | 41736456 | 41743229 |
| *BvbZIP46* | LOC104884020 | A | scaffold00419 | 5540 | 16678 |
| *BvbZIP47* | LOC104884843 | D | scaffold00899 | 52251 | 61547 |
| *BvbZIP48* | LOC104884708 | G | scaffold00796 | 83055 | 94429 |
| *AhbZIP1* | AHYPO_000803 | S | scaffold_3 | 1090676 | 1091164 |
| *AhbZIP2* | AHYPO_001450 | S | scaffold_7 | 462759 | 463184 |
| *AhbZIP3* | AHYPO_002095 | S | scaffold_10 | 1356708 | 1357536 |
| *AhbZIP4* | AHYPO_002307 | E | scaffold_12 | 345407 | 352110 |
| *AhbZIP5* | AHYPO_002802 | A | scaffold_15 | 1266014 | 1269169 |
| *AhbZIP6* | AHYPO_003073 | S | scaffold_17 | 1174756 | 1175310 |
| *AhbZIP7* | AHYPO_003169 | S | scaffold_18 | 912436 | 912948 |
| *AhbZIP8* | AHYPO_003221 | C | scaffold_19 | 89886 | 96897 |
| *AhbZIP9* | AHYPO_003399 | C | scaffold_20 | 775388 | 786759 |
| *AhbZIP10* | AHYPO_003681 | F | scaffold_22 | 1071041 | 1075750 |
| *AhbZIP11* | AHYPO_003736 | I | scaffold_23 | 256625 | 259657 |
| *AhbZIP12* | AHYPO_005263 | G | scaffold_35 | 401980 | 405447 |
| *AhbZIP13* | AHYPO_005409 | D | scaffold_36 | 631853 | 648789 |
| *AhbZIP14* | AHYPO_005567 | D | scaffold_38 | 172371 | 178214 |
| *AhbZIP15* | AHYPO_005619 | S | scaffold_38 | 760960 | 761678 |
| *AhbZIP16* | AHYPO_005775 | I | scaffold_40 | 510617 | 519779 |
| *AhbZIP17* | AHYPO_005861 | S | scaffold_41 | 633325 | 633867 |
| *AhbZIP18* | AHYPO_006154 | B | scaffold_44 | 697328 | 700322 |
| *AhbZIP19* | AHYPO_006289 | A | scaffold_46 | 49678 | 55138 |
| *AhbZIP20* | AHYPO_007596 | A | scaffold_61 | 426326 | 432853 |
| *AhbZIP21* | AHYPO_008855 | D | scaffold_80 | 26022 | 33292 |
| *AhbZIP22* | AHYPO_008893 | B | scaffold_80 | 420557 | 424412 |
| *AhbZIP23* | AHYPO_008948 | S | scaffold_81 | 225718 | 226143 |
| *AhbZIP24* | AHYPO_009646 | C | scaffold_92 | 664205 | 669942 |
| *AhbZIP25* | AHYPO_009973 | A | scaffold_96 | 649139 | 652059 |
| *AhbZIP26* | AHYPO_010724 | S | scaffold_110 | 113784 | 114668 |
| *AhbZIP27* | AHYPO_010774 | S | scaffold_111 | 287103 | 287600 |
| *AhbZIP28* | AHYPO_011169 | I | scaffold_118 | 429348 | 439529 |
| *AhbZIP29* | AHYPO_011258 | I | scaffold_120 | 261903 | 269085 |
| *AhbZIP30* | AHYPO_011539 | E | scaffold_127 | 17942 | 21761 |
| *AhbZIP31* | AHYPO_011703 | A | scaffold_130 | 577037 | 580845 |
| *AhbZIP32* | AHYPO_011891 | S | scaffold_135 | 225033 | 225586 |
| *AhbZIP33* | AHYPO_012318 | F | scaffold_146 | 393420 | 394271 |
| *AhbZIP34* | AHYPO_012724 | S | scaffold_156 | 356304 | 356853 |
| *AhbZIP35* | AHYPO_013439 | I | scaffold_179 | 4224 | 7700 |
| *AhbZIP36* | AHYPO_013738 | E | scaffold_188 | 238311 | 240390 |
| *AhbZIP37* | AHYPO_014108 | H | scaffold_203 | 21936 | 26140 |
| *AhbZIP38* | AHYPO_014909 | D | scaffold_235 | 247409 | 257635 |
| *AhbZIP39* | AHYPO_015040 | D | scaffold_241 | 304344 | 311250 |
| *AhbZIP40* | AHYPO_015644 | G | scaffold_270 | 206484 | 213212 |
| *AhbZIP41* | AHYPO_015995 | S | scaffold_288 | 172948 | 173202 |
| *AhbZIP42* | AHYPO_017405 | H | scaffold_384 | 9753 | 14327 |
| *AhbZIP43* | AHYPO_017540 | I | scaffold_392 | 188716 | 192252 |
| *AhbZIP44* | AHYPO_017835 | E | scaffold_418 | 110296 | 116908 |
| *AhbZIP45* | AHYPO_017955 | D | scaffold_430 | 66009 | 71982 |
| *AhbZIP46* | AHYPO_019738 | S | scaffold_630 | 92481 | 93218 |
| *AhbZIP47* | AHYPO_020532 | K | scaffold_773 | 22805 | 26614 |
| *AhbZIP48* | AHYPO_021039 | F | scaffold_893 | 76005 | 76669 |
| *AhbZIP49* | AHYPO_021226 | S | scaffold_935 | 12014 | 12535 |
