# Supplementary material for: Genome-wide survey, characterization, and expression analysis of bZIP transcription factors in Chenopodium quinoa
Source: BMC Plant Biol. 2020 Sep 1;20:405. doi: 10.1186/s12870-020-02620-z (PMC7466520; doi:10.1186/s12870-020-02620-z)
Supplement: Supplementary file 3 — Additional file 3. The classification and gene structures of bZIPs in quinoa. [file 12870_2020_2620_MOESM3_ESM.doc]

**Additional file 3:** The classification and gene structures of *bZIPs* in quinoa

| Name | Subfamily | Exon number | Intron number |
| --- | --- | --- | --- |
| *CqbZIP1* | A | 3 | 2 |
| *CqbZIP2* | C | 6 | 5 |
| *CqbZIP3* | A | 6 | 5 |
| *CqbZIP4* | D | 8 | 7 |
| *CqbZIP5* | I | 4 | 3 |
| *CqbZIP6* | H | 4 | 3 |
| *CqbZIP7* | D | 11 | 10 |
| *CqbZIP8* | G | 11 | 10 |
| *CqbZIP9* | D | 8 | 7 |
| *CqbZIP10* | S | 1 | 0 |
| *CqbZIP11* | S | 1 | 0 |
| *CqbZIP12* | G | 12 | 11 |
| *CqbZIP13* | E | 4 | 3 |
| *CqbZIP14* | F | 1 | 0 |
| *CqbZIP15* | C | 6 | 5 |
| *CqbZIP16* | S | 1 | 0 |
| *CqbZIP17* | A | 5 | 4 |
| *CqbZIP18* | A | 3 | 2 |
| *CqbZIP19* | G | 12 | 11 |
| *CqbZIP20* | S | 2 | 1 |
| *CqbZIP21* | S | 1 | 0 |
| *CqbZIP22* | S | 1 | 0 |
| *CqbZIP23* | S | 1 | 0 |
| *CqbZIP24* | G | 11 | 10 |
| *CqbZIP25* | S | 1 | 0 |
| *CqbZIP26* | I | 4 | 3 |
| *CqbZIP27* | D | 9 | 8 |
| *CqbZIP28* | A | 6 | 5 |
| *CqbZIP29* | F | 3 | 2 |
| *CqbZIP30* | I | 5 | 4 |
| *CqbZIP31* | A | 1 | 0 |
| *CqbZIP32* | S | 1 | 0 |
| *CqbZIP33* | S | 1 | 0 |
| *CqbZIP34* | F | 1 | 0 |
| *CqbZIP35* | I | 5 | 4 |
| *CqbZIP36* | A | 2 | 1 |
| *CqbZIP37* | I | 4 | 3 |
| *CqbZIP38* | No group | 8 | 7 |
| *CqbZIP39* | F | 1 | 0 |
| *CqbZIP40* | S | 1 | 0 |
| *CqbZIP41* | I | 4 | 3 |
| *CqbZIP42* | A | 3 | 2 |
| *CqbZIP43* | D | 11 | 10 |
| *CqbZIP44* | B | 5 | 4 |
| *CqbZIP45* | A | 3 | 2 |
| *CqbZIP46* | G | 11 | 10 |
| *CqbZIP47* | I | 6 | 5 |
| *CqbZIP48* | C | 5 | 4 |
| *CqbZIP49* | A | 3 | 2 |
| *CqbZIP50* | G | 11 | 10 |
| *CqbZIP51* | S | 2 | 1 |
| *CqbZIP52* | D | 8 | 7 |
| *CqbZIP53* | S | 1 | 0 |
| *CqbZIP54* | G | 12 | 11 |
| *CqbZIP55* | E | 4 | 3 |
| *CqbZIP56* | H | 5 | 4 |
| *CqbZIP57* | I | 3 | 2 |
| *CqbZIP58* | E | 5 | 4 |
| *CqbZIP59* | A | 1 | 0 |
| *CqbZIP60* | S | 1 | 0 |
| *CqbZIP61* | H | 4 | 3 |
| *CqbZIP62* | I | 5 | 4 |
| *CqbZIP63* | A | 5 | 4 |
| *CqbZIP64* | I | 6 | 5 |
| *CqbZIP65* | A | 4 | 3 |
| *CqbZIP66* | J | 5 | 4 |
| *CqbZIP67* | B | 5 | 4 |
| *CqbZIP68* | D | 10 | 9 |
| *CqbZIP69* | I | 5 | 4 |
| *CqbZIP70* | H | 3 | 2 |
| *CqbZIP71* | G | 11 | 10 |
| *CqbZIP72* | D | 11 | 10 |
| *CqbZIP73* | D | 11 | 10 |
| *CqbZIP74* | No group | 9 | 8 |
| *CqbZIP75* | J | 6 | 5 |
| *CqbZIP76* | F | 1 | 0 |
| *CqbZIP77* | F | 1 | 0 |
| *CqbZIP78* | E | 4 | 3 |
| *CqbZIP79* | E | 4 | 3 |
| *CqbZIP80* | S | 1 | 0 |
| *CqbZIP81* | K | 4 | 3 |
| *CqbZIP82* | D | 11 | 10 |
| *CqbZIP83* | No group | 8 | 7 |
| *CqbZIP84* | I | 4 | 3 |
| *CqbZIP85* | F | 4 | 3 |
| *CqbZIP86* | No group | 8 | 7 |
| *CqbZIP87* | C | 5 | 4 |
| *CqbZIP88* | E | 5 | 4 |
| *CqbZIP89* | D | 11 | 10 |
| *CqbZIP90* | G | 4 | 3 |
| *CqbZIP91* | F | 1 | 0 |
| *CqbZIP92* | K | 3 | 2 |
| *CqbZIP93* | S | 1 | 0 |
| *CqbZIP94* | D | 8 | 7 |
