# Supplementary material for: Genome-wide survey, characterization, and expression analysis of bZIP transcription factors in Chenopodium quinoa
Source: BMC Plant Biol. 2020 Sep 1;20:405. doi: 10.1186/s12870-020-02620-z (PMC7466520; doi:10.1186/s12870-020-02620-z)
Supplement: Supplementary file 4 — Additional file 4. The percentage of members in each bZIP subfamily in quinoa, spinach, sugar beet, and amaranth. [file 12870_2020_2620_MOESM4_ESM.pdf]

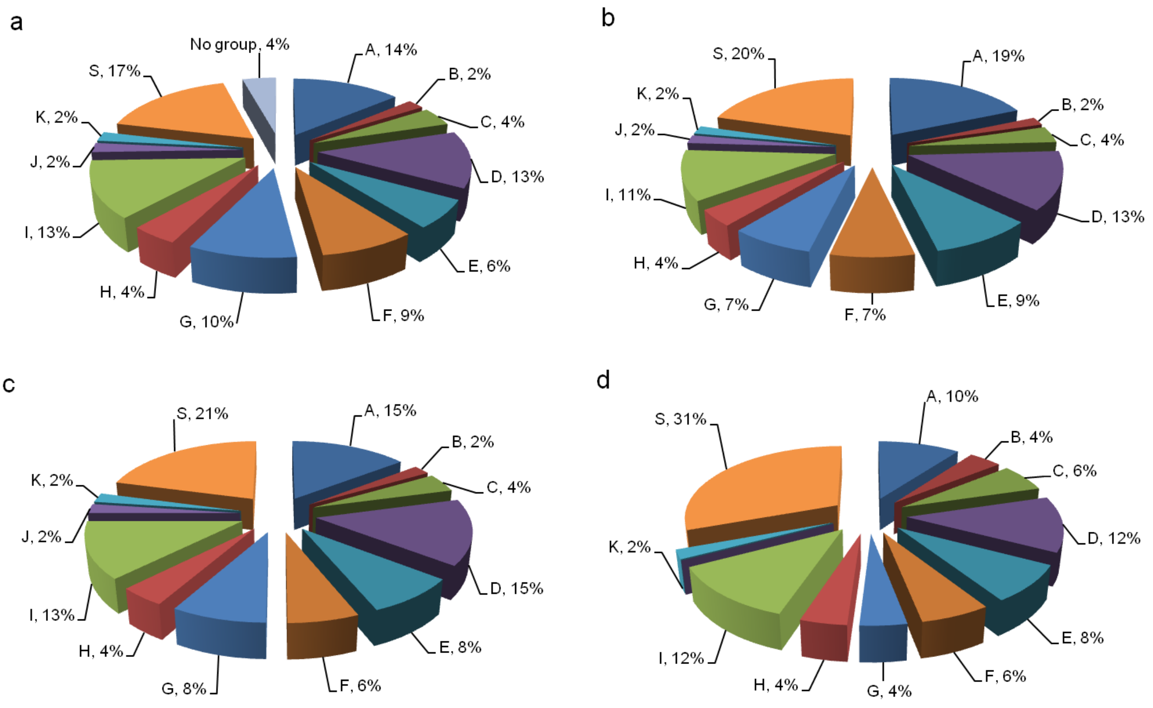

Additional file 4: The percentage of members in each *bZIP* subfamily in quinoa (**a**), spinach (**b**), sugar beet (**c**), and amaranth (**d**).
