# Supplementary figures and images for: Genome-wide survey, characterization, and expression analysis of bZIP transcription factors in Chenopodium quinoa
Source: BMC Plant Biol. 2020 Sep 1;20:405. doi: 10.1186/s12870-020-02620-z (PMC7466520; doi:10.1186/s12870-020-02620-z)

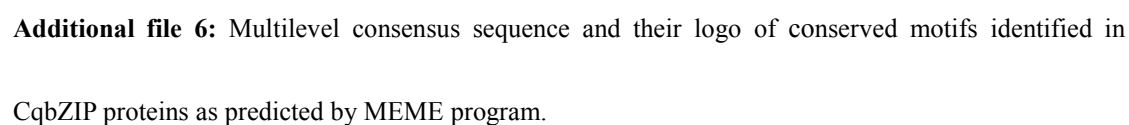

CqbZIP proteins as predicted by MEME program.

Supplement: Supplementary file 6 — Additional file 6. Multilevel consensus sequence and their logo of conserved motifs identified in CqbZIP proteins as predicted by MEME program. [file 12870_2020_2620_MOESM6_ESM.pdf]
