# Supplementary material for: Genome-wide survey, characterization, and expression analysis of bZIP transcription factors in Chenopodium quinoa
Source: BMC Plant Biol. 2020 Sep 1;20:405. doi: 10.1186/s12870-020-02620-z (PMC7466520; doi:10.1186/s12870-020-02620-z)
Supplement: Supplementary file 7 — Additional file 7. Genomic locations of bZIPs in quinoa. [file 12870_2020_2620_MOESM7_ESM.doc]

**Additional file 7:** Genomic locations of *bZIPs* in quinoa

| Name | Chromosome Name | Gene Start (bp) | Gene End (bp) | Gene Orientation |
| --- | --- | --- | --- | --- |
| *CqbZIP1* | C_Quinoa_Scaffold_2088 | 4578769 | 4584238 | Reverse |
| *CqbZIP2* | C_Quinoa_Scaffold_2088 | 12375441 | 12385410 | Reverse |
| *CqbZIP3* | C_Quinoa_Scaffold_2716 | 4257413 | 4260892 | Reverse |
| *CqbZIP4* | C_Quinoa_Scaffold_4480 | 1621471 | 1625526 | Forward |
| *CqbZIP5* | C_Quinoa_Scaffold_4480 | 5337228 | 5344160 | Forward |
| *CqbZIP6* | C_Quinoa_Scaffold_4480 | 8021189 | 8027527 | Reverse |
| *CqbZIP7* | C_Quinoa_Scaffold_1000 | 15594465 | 15598799 | Reverse |
| *CqbZIP8* | C_Quinoa_Scaffold_1000 | 20929017 | 20936324 | Reverse |
| *CqbZIP9* | C_Quinoa_Scaffold_2370 | 4193566 | 4198135 | Reverse |
| *CqbZIP10* | C_Quinoa_Scaffold_2370 | 7568620 | 7569630 | Reverse |
| *CqbZIP11* | C_Quinoa_Scaffold_2370 | 8466288 | 8466830 | Forward |
| *CqbZIP12* | C_Quinoa_Scaffold_4250 | 3173484 | 3178356 | Forward |
| *CqbZIP13* | C_Quinoa_Scaffold_4250 | 5528888 | 5530454 | Forward |
| *CqbZIP14* | C_Quinoa_Scaffold_3820 | 261741 | 262484 | Forward |
| *CqbZIP15* | C_Quinoa_Scaffold_3820 | 7210948 | 7222047 | Forward |
| *CqbZIP16* | C_Quinoa_Scaffold_1001 | 949159 | 949686 | Reverse |
| *CqbZIP17* | C_Quinoa_Scaffold_1001 | 3389620 | 3396918 | Forward |
| *CqbZIP18* | C_Quinoa_Scaffold_3429 | 4993222 | 4998435 | Reverse |
| *CqbZIP19* | C_Quinoa_Scaffold_1971 | 4524274 | 4533956 | Reverse |
| *CqbZIP20* | C_Quinoa_Scaffold_2646 | 170124 | 171126 | Reverse |
| *CqbZIP21* | C_Quinoa_Scaffold_2646 | 970573 | 971112 | Forward |
| *CqbZIP22* | C_Quinoa_Scaffold_2646 | 4757545 | 4758036 | Reverse |
| *CqbZIP23* | C_Quinoa_Scaffold_2646 | 4790565 | 4791065 | Reverse |
| *CqbZIP24* | C_Quinoa_Scaffold_2646 | 7423417 | 7431418 | Reverse |
| *CqbZIP25* | C_Quinoa_Scaffold_3674 | 5340588 | 5341142 | Reverse |
| *CqbZIP26* | C_Quinoa_Scaffold_2493 | 2698741 | 2701739 | Reverse |
| *CqbZIP27* | C_Quinoa_Scaffold_2493 | 2978878 | 2985687 | Forward |
| *CqbZIP28* | C_Quinoa_Scaffold_2493 | 5096126 | 5099629 | Forward |
| *CqbZIP29* | C_Quinoa_Scaffold_1257 | 1031405 | 1035730 | Reverse |
| *CqbZIP30* | C_Quinoa_Scaffold_1257 | 4399681 | 4402547 | Reverse |
| *CqbZIP31* | C_Quinoa_Scaffold_2008 | 1251572 | 1251850 | Reverse |
| *CqbZIP32* | C_Quinoa_Scaffold_2008 | 1258647 | 1259072 | Reverse |
| *CqbZIP33* | C_Quinoa_Scaffold_2715 | 3091098 | 3091718 | Reverse |
| *CqbZIP34* | C_Quinoa_Scaffold_1040 | 1501208 | 1501957 | Reverse |
| *CqbZIP35* | C_Quinoa_Scaffold_1611 | 5020069 | 5027928 | Reverse |
| *CqbZIP36* | C_Quinoa_Scaffold_1412 | 4634208 | 4634910 | Forward |
| *CqbZIP37* | C_Quinoa_Scaffold_1566 | 2651795 | 2657186 | Reverse |
| *CqbZIP38* | C_Quinoa_Scaffold_1566 | 4135762 | 4140067 | Reverse |
| *CqbZIP39* | C_Quinoa_Scaffold_1566 | 4540890 | 4541702 | Forward |
| *CqbZIP40* | C_Quinoa_Scaffold_3256 | 2013084 | 2013578 | Reverse |
| *CqbZIP41* | C_Quinoa_Scaffold_3670 | 2735463 | 2739265 | Reverse |
| *CqbZIP42* | C_Quinoa_Scaffold_2048 | 9506428 | 9509751 | Reverse |
| *CqbZIP43* | C_Quinoa_Scaffold_4077 | 231615 | 239778 | Forward |
| *CqbZIP44* | C_Quinoa_Scaffold_4077 | 915211 | 918503 | Forward |
| *CqbZIP45* | C_Quinoa_Scaffold_4077 | 2079850 | 2083062 | Reverse |
| *CqbZIP46* | C_Quinoa_Scaffold_1817 | 322234 | 331532 | Forward |
| *CqbZIP47* | C_Quinoa_Scaffold_3876 | 3509317 | 3515659 | Reverse |
| *CqbZIP48* | C_Quinoa_Scaffold_3966 | 1515087 | 1516920 | Reverse |
| *CqbZIP49* | C_Quinoa_Scaffold_4257 | 1865388 | 1868736 | Forward |
| *CqbZIP50* | C_Quinoa_Scaffold_1783 | 153948 | 159959 | Reverse |
| *CqbZIP51* | C_Quinoa_Scaffold_1862 | 1823904 | 1826018 | Forward |
| *CqbZIP52* | C_Quinoa_Scaffold_3163 | 464383 | 470668 | Reverse |
| *CqbZIP53* | C_Quinoa_Scaffold_1189 | 1574616 | 1575170 | Reverse |
| *CqbZIP54* | C_Quinoa_Scaffold_1606 | 798840 | 803611 | Reverse |
| *CqbZIP55* | C_Quinoa_Scaffold_1606 | 921162 | 922608 | Forward |
| *CqbZIP56* | C_Quinoa_Scaffold_2187 | 496245 | 506480 | Forward |
| *CqbZIP57* | C_Quinoa_Scaffold_2187 | 2975465 | 2980817 | Reverse |
| *CqbZIP58* | C_Quinoa_Scaffold_3389 | 5392678 | 5394879 | Reverse |
| *CqbZIP59* | C_Quinoa_Scaffold_1992 | 1287292 | 1287630 | Reverse |
| *CqbZIP60* | C_Quinoa_Scaffold_1992 | 1294091 | 1294516 | Reverse |
| *CqbZIP61* | C_Quinoa_Scaffold_1516 | 2382576 | 2386813 | Forward |
| *CqbZIP62* | C_Quinoa_Scaffold_1516 | 2922062 | 2924360 | Reverse |
| *CqbZIP63* | C_Quinoa_Scaffold_2081 | 3248095 | 3254844 | Forward |
| *CqbZIP64* | C_Quinoa_Scaffold_2081 | 4000480 | 4009971 | Forward |
| *CqbZIP65* | C_Quinoa_Scaffold_3100 | 6005675 | 6008201 | Reverse |
| *CqbZIP66* | C_Quinoa_Scaffold_3100 | 6869974 | 6871646 | Reverse |
| *CqbZIP67* | C_Quinoa_Scaffold_3970 | 177414 | 180779 | Reverse |
| *CqbZIP68* | C_Quinoa_Scaffold_3970 | 895506 | 902887 | Reverse |
| *CqbZIP69* | C_Quinoa_Scaffold_3631 | 1207621 | 1209840 | Forward |
| *CqbZIP70* | C_Quinoa_Scaffold_3631 | 1601334 | 1603972 | Reverse |
| *CqbZIP71* | C_Quinoa_Scaffold_2963 | 2668090 | 2673812 | Forward |
| *CqbZIP72* | C_Quinoa_Scaffold_1695 | 2799907 | 2807648 | Reverse |
| *CqbZIP73* | C_Quinoa_Scaffold_1251 | 1578542 | 1586429 | Reverse |
| *CqbZIP74* | C_Quinoa_Scaffold_4206 | 5870214 | 5888641 | Reverse |
| *CqbZIP75* | C_Quinoa_Scaffold_1776 | 533159 | 535902 | Reverse |
| *CqbZIP76* | C_Quinoa_Scaffold_2123 | 1518465 | 1519049 | Forward |
| *CqbZIP77* | C_Quinoa_Scaffold_2123 | 1558402 | 1559031 | Forward |
| *CqbZIP78* | C_Quinoa_Scaffold_2123 | 3714924 | 3720781 | Forward |
| *CqbZIP79* | C_Quinoa_Scaffold_1522 | 269627 | 275043 | Forward |
| *CqbZIP80* | C_Quinoa_Scaffold_1522 | 2052520 | 2053086 | Forward |
| *CqbZIP81* | C_Quinoa_Scaffold_2624 | 1248050 | 1249723 | Reverse |
| *CqbZIP82* | C_Quinoa_Scaffold_2868 | 2133954 | 2138511 | Reverse |
| *CqbZIP83* | C_Quinoa_Scaffold_1759 | 212839 | 222365 | Reverse |
| *CqbZIP84* | C_Quinoa_Scaffold_2528 | 317072 | 319904 | Forward |
| *CqbZIP85* | C_Quinoa_Scaffold_4036 | 1996730 | 2002589 | Reverse |
| *CqbZIP86* | C_Quinoa_Scaffold_3859 | 639478 | 647026 | Reverse |
| *CqbZIP87* | C_Quinoa_Scaffold_3144 | 419550 | 422225 | Forward |
| *CqbZIP88* | C_Quinoa_Scaffold_1059 | 7180535 | 7182706 | Reverse |
| *CqbZIP89* | C_Quinoa_Scaffold_2771 | 25460 | 33598 | Forward |
| *CqbZIP90* | C_Quinoa_Scaffold_4462 | 56358 | 62090 | Reverse |
| *CqbZIP91* | C_Quinoa_Scaffold_2371 | 683246 | 683875 | Forward |
| *CqbZIP92* | C_Quinoa_Scaffold_3745 | 342286 | 345169 | Reverse |
| *CqbZIP93* | C_Quinoa_Scaffold_2159 | 97534 | 98031 | Reverse |
| *CqbZIP94* | C_Quinoa_Scaffold_1108 | 111143 | 117347 | Forward |
