# Supplementary material for: Genome-wide survey, characterization, and expression analysis of bZIP transcription factors in Chenopodium quinoa
Source: BMC Plant Biol. 2020 Sep 1;20:405. doi: 10.1186/s12870-020-02620-z (PMC7466520; doi:10.1186/s12870-020-02620-z)
Supplement: Supplementary file 8 — Additional file 8. Ka/Ks analysis for orthologous bZIP gene pairs between quinoa and spinach, sugar beet, and amaranth. [file 12870_2020_2620_MOESM8_ESM.doc]

**Additional file 8:** Ka/Ks analysis for orthologous *bZIP* gene pairs between quinoa and spinach, beet, and amaranth

|  | Orthologous gene pairs | | Subfamily | Ka | Ks | Ka/Ks | Purifing selection |
| --- | --- | --- | --- | --- | --- | --- | --- |
| *SobZIPs*  vs  *CqbZIPs* | *SobZIP1* | *CqbZIP54* | G | 0.0811 | 0.3035 | 0.2672 | Yes |
|  |  | *CqbZIP12* | G | 0.0864 | 0.3581 | 0.2413 | Yes |
|  | *SobZIP2* | *CqbZIP19* | G | 0.0684 | 0.2939 | 0.2327 | Yes |
|  |  | *CqbZIP46* | G | 0.0637 | 0.3183 | 0.2001 | Yes |
|  | *SobZIP4* | *CqbZIP48* | C | 0.1201 | 0.4886 | 0.2458 | Yes |
|  |  | *CqbZIP87* | C | 0.1132 | 0.6041 | 0.1874 | Yes |
|  | *SobZIP5* | *CqbZIP32* | S | 0.0492 | 0.6463 | 0.0761 | Yes |
|  |  | *CqbZIP60* | S | 0.0493 | 0.7225 | 0.0682 | Yes |
|  | *SobZIP6* | *CqbZIP50* | G | 0.0807 | 0.3105 | 0.2599 | Yes |
|  |  | *CqbZIP71* | G | 0.1483 | 0.2740 | 0.5412 | Yes |
|  | *SobZIP11* | *CqbZIP4* | D | 0.0275 | 0.4237 | 0.0649 | Yes |
|  |  | *CqbZIP9* | D | 0.0268 | 0.4375 | 0.0613 | Yes |
|  | *SobZIP15* | *CqbZIP52* | D | 0.0599 | 0.3601 | 0.1663 | Yes |
|  |  | *CqbZIP94* | D | 0.0709 | 0.3453 | 0.2053 | Yes |
|  | *SobZIP18* | *CqbZIP41* | I | 0.0521 | 0.5030 | 0.1036 | Yes |
|  |  | *CqbZIP30* | I | 0.0638 | 0.4654 | 0.1371 | Yes |
|  | *SobZIP22* | *CqbZIP5* | I | 0.1169 | 0.4467 | 0.2617 | Yes |
|  |  | *CqbZIP57* | I | 0.0619 | 0.4151 | 0.1491 | Yes |
|  | *SobZIP23* | *CqbZIP43* | D | 0.0344 | 0.2825 | 0.1218 | Yes |
|  |  | *CqbZIP68* | D | 0.0322 | 0.2684 | 0.1200 | Yes |
|  | *SobZIP30* | *CqbZIP26* | I | 0.0592 | 0.4890 | 0.1211 | Yes |
|  |  | *CqbZIP84* | I | 0.0585 | 0.4049 | 0.1445 | Yes |
|  | *SobZIP31* | *CqbZIP73* | D | 0.0214 | 0.3532 | 0.0606 | Yes |
|  |  | *CqbZIP72* | D | 0.0224 | 0.3059 | 0.0732 | Yes |
|  | *SobZIP37* | *CqbZIP7* | D | 0.0646 | 0.3717 | 0.1738 | Yes |
|  |  | *CqbZIP82* | D | 0.0635 | 0.3755 | 0.1691 | Yes |
|  | *SobZIP39* | *CqbZIP2* | C | 0.0998 | 0.3716 | 0.2686 | Yes |
|  |  | *CqbZIP15* | C | 0.1019 | 0.3494 | 0.2916 | Yes |
|  | *SobZIP41* | *CqbZIP8* | G | 0.0556 | 0.2837 | 0.1960 | Yes |
|  |  | *CqbZIP24* | G | 0.0583 | 0.2895 | 0.2014 | Yes |
|  | *SobZIP43* | *CqbZIP49* | A | 0.0483 | 0.3312 | 0.1458 | Yes |
|  |  | *CqbZIP45* | A | 0.0490 | 0.3649 | 0.1343 | Yes |
|  | *SobZIP45* | *CqbZIP25* | S | 0.0500 | 0.3032 | 0.1649 | Yes |
|  |  | *CqbZIP53* | S | 0.0557 | 0.2802 | 0.1988 | Yes |
|  | *SobZIP3* | *CqbZIP16* | S | 0.0431 | 0.5389 | 0.0800 | Yes |
|  | *SobZIP12* | *CqbZIP39* | F | 0.0724 | 0.3323 | 0.2179 | Yes |
|  | *SobZIP32* | *CqbZIP27* | D | 0.0194 | 0.4240 | 0.0458 | Yes |
|  | *SobZIP34* | *CqbZIP29* | F | 0.0417 | 0.4033 | 0.1034 | Yes |
|  | *SobZIP38* | *CqbZIP6* | H | 0.1508 | 0.3982 | 0.3787 | Yes |
|  | *SobZIP42* | *CqbZIP65* | A | 0.0421 | 0.5056 | 0.0833 | Yes |
|  | *SobZIP50* | *CqbZIP61* | H | 0.0248 | 0.3399 | 0.0730 | Yes |
| *BvbZIPs*  vs  *CqbZIPs* | *BvbZIP1* | *CqbZIP52* | D | 0.1118 | 0.3989 | 0.2803 | Yes |
|  |  | *CqbZIP94* | D | 0.1120 | 0.3553 | 0.3152 | Yes |
|  | *BvbZIP11* | *CqbZIP25* | S | 0.0653 | 0.4556 | 0.1433 | Yes |
|  |  | *CqbZIP53* | S | 0.0652 | 0.3982 | 0.1637 | Yes |
|  | *BvbZIP13* | *CqbZIP73* | D | 0.1540 | 0.0779 | 1.9769 | No |
|  |  | *CqbZIP72* | D | 0.1494 | 0.0779 | 1.9178 | No |
|  | *BvbZIP19* | *CqbZIP5* | I | 0.1167 | 0.7118 | 0.1640 | Yes |
|  |  | *CqbZIP57* | I | 0.0638 | 0.6645 | 0.0960 | Yes |
|  | *BvbZIP21* | *CqbZIP43* | D | 0.0554 | 0.3260 | 0.1699 | Yes |
|  |  | *CqbZIP68* | D | 0.0492 | 0.3282 | 0.1499 | Yes |
|  | *BvbZIP23* | *CqbZIP49* | A | 0.0398 | 0.4049 | 0.0983 | Yes |
|  |  | *CqbZIP45* | A | 0.0456 | 0.4692 | 0.0972 | Yes |
|  | *BvbZIP27* | *CqbZIP19* | G | 0.0651 | 0.3267 | 0.1993 | Yes |
|  |  | *CqbZIP46* | G | 0.0603 | 0.3759 | 0.1604 | Yes |
|  | *BvbZIP28* | *CqbZIP32* | S | 0.0697 | 0.8353 | 0.0834 | Yes |
|  |  | *CqbZIP60* | S | 0.0698 | 0.8974 | 0.0778 | Yes |
|  | *BvbZIP32* | *CqbZIP4* | D | 0.0600 | 0.5287 | 0.1135 | Yes |
|  |  | *CqbZIP9* | D | 0.0593 | 0.5274 | 0.1124 | Yes |
|  | *BvbZIP38* | *CqbZIP26* | I | 0.1083 | 0.6700 | 0.1616 | Yes |
|  |  | *CqbZIP84* | I | 0.0951 | 0.5324 | 0.1786 | Yes |
|  | *BvbZIP41* | *CqbZIP41* | I | 0.0608 | 0.5473 | 0.1111 | Yes |
|  |  | *CqbZIP30* | I | 0.0688 | 0.5176 | 0.1329 | Yes |
|  | *BvbZIP47* | *CqbZIP7* | D | 0.0540 | 0.3837 | 0.1407 | Yes |
|  |  | *CqbZIP82* | D | 0.1059 | 0.4399 | 0.2407 | Yes |
|  | *BvbZIP48* | *CqbZIP8* | G | 0.0532 | 0.4122 | 0.1291 | Yes |
|  |  | *CqbZIP24* | G | 0.0559 | 0.3765 | 0.1485 | Yes |
|  | *BvbZIP3* | *CqbZIP16* | S | 0.0588 | 0.5366 | 0.1096 | Yes |
|  | *BvbZIP15* | *CqbZIP61* | H | 0.0371 | 0.4695 | 0.0790 | Yes |
|  | *BvbZIP16* | *CqbZIP56* | H | 0.1309 | 0.7019 | 0.1865 | Yes |
|  | *BvbZIP34* | *CqbZIP22* | S | 0.1191 | 0.5254 | 0.2267 | Yes |
|  | *BvbZIP39* | *CqbZIP27* | D | 0.0506 | 0.6527 | 0.0775 | Yes |
|  | *BvbZIP44* | *CqbZIP29* | F | 0.0755 | 0.6558 | 0.1151 | Yes |
| *AhbZIPs*  vs  *CqbZIPs* | *AhbZIP6* | *CqbZIP53* | S | 0.0819 | 0.4619 | 0.1773 | Yes |
|  |  | *CqbZIP25* | S | 0.0833 | 0.4835 | 0.1723 | Yes |
|  | *AhbZIP21* | *CqbZIP43* | D | 0.0664 | 0.5261 | 0.1262 | Yes |
|  |  | *CqbZIP68* | D | 0.0663 | 0.5275 | 0.1257 | Yes |
|  | *AhbZIP23* | *CqbZIP32* | S | 0.0848 | 1.5642 | 0.0542 | Yes |
|  |  | *CqbZIP60* | S | 0.0749 | 2.1664 | 0.0346 | Yes |
|  | *AhbZIP28* | *CqbZIP57* | I | 0.0482 | 0.7058 | 0.0683 | Yes |
|  |  | *CqbZIP5* | I | 0.0987 | 0.7566 | 0.1305 | Yes |
|  | *AhbZIP39* | *CqbZIP72* | D | 0.0631 | 0.4405 | 0.1432 | Yes |
|  |  | *CqbZIP73* | D | 0.0623 | 0.4569 | 0.1364 | Yes |
|  | *AhbZIP43* | *CqbZIP41* | I | 0.0886 | 0.7068 | 0.1254 | Yes |
|  |  | *CqbZIP30* | I | 0.0932 | 0.6935 | 0.1344 | Yes |
|  | *AhbZIP45* | *CqbZIP9* | D | 0.0831 | 0.6201 | 0.1340 | Yes |
|  |  | *CqbZIP4* | D | 0.0886 | 0.5799 | 0.1528 | Yes |
|  | *AhbZIP10* | *CqbZIP29* | F | 0.1077 | 0.7182 | 0.1500 | Yes |
|  | *AhbZIP42* | *CqbZIP61* | H | 0.0419 | 0.4931 | 0.0850 | Yes |
