# Supplementary material for: Genome-wide survey, characterization, and expression analysis of bZIP transcription factors in Chenopodium quinoa
Source: BMC Plant Biol. 2020 Sep 1;20:405. doi: 10.1186/s12870-020-02620-z (PMC7466520; doi:10.1186/s12870-020-02620-z)
Supplement: Supplementary file 9 — Additional file 9. Orthology information of CqbZIPs and AtbZIPs from BLASTP. [file 12870_2020_2620_MOESM9_ESM.doc]

**Additional file 9:** Orthology information of CqbZIPs and AtbZIPs from BLASTP

| CqbZIP Name | AtbZIP Name | Max Score | Total Score | Query Cover | E-Value | Per.ident |
| --- | --- | --- | --- | --- | --- | --- |
| CqbZIP3 | ABF2/AT1G45249 | 193.0 | 323.0 | 0.88 | 7.00E-58 | 60.10% |
| CqbZIP3 | ABF4/AT3G19290 | 140.0 | 276.0 | 0.84 | 8.00E-38 | 54.61% |
| CqbZIP3 | ABF1/ AT1G49720 | 136.0 | 245.0 | 0.74 | 7.00E-36 | 52.29% |
| CqbZIP3 | ABF3/AT4G34000 | 130.0 | 253.0 | 0.86 | 8.00E-34 | 50.00% |
| CqbZIP8 | GBF1/ AT4G36730 | 318.0 | 318.0 | 0.99 | 6.00E-107 | 50.40% |
| CqbZIP24 | GBF1/ AT4G36730 | 313.0 | 313.0 | 0.99 | 2.00E-105 | 50.00% |
| CqbZIP44 | bZIP17/AT2G40950 | 380.0 | 380.0 | 0.74 | 2.00E-121 | 48.42% |
| CqbZIP44 | bZIP49/ AT3G56660 | 327.0 | 327.0 | 0.74 | 3.00E-102 | 41.68% |
| CqbZIP44 | bZIP28/ AT3G10800 | 303.0 | 303.0 | 0.79 | 2.00E-92 | 39.71% |
| CqbZIP67 | bZIP17/AT2G40950 | 399.0 | 399.0 | 0.97 | 4.00E-129 | 45.09% |
| CqbZIP67 | bZIP49/ AT3G56660 | 345.0 | 345.0 | 0.75 | 1.00E-109 | 44.49% |
| CqbZIP67 | bZIP28/ AT3G10800 | 319.0 | 319.0 | 0.83 | 5.00E-99 | 41.78% |
| CqbZIP81 | bZIP60/AT1G42990 | 71.6 | 71.6 | 0.68 | 6.00E-14 | 41.67% |
| CqbZIP92 | bZIP60/AT1G42990 | 72.0 | 72.0 | 0.60 | 6.00E-14 | 42.19% |
| CqbZIP72 | TGA6/AT3G12250 | 510.0 | 510.0 | 0.71 | 0.00E+00 | 79.22% |
| CqbZIP72 | TGA2/AT5G06950 | 489.0 | 489.0 | 0.71 | 3.00E-173 | 79.22% |
| CqbZIP72 | TGA5/AT5G06960 | 468.0 | 468.0 | 0.71 | 1.00E-164 | 75.60% |
| CqbZIP73 | TGA6/AT3G12250 | 532.0 | 532.0 | 0.72 | 0.00E+00 | 81.33% |
| CqbZIP73 | TGA2/AT5G06950 | 512.0 | 512.0 | 0.72 | 0.00E+00 | 81.33% |
| CqbZIP73 | TGA5/AT5G06960 | 484.0 | 484.0 | 0.72 | 5.00E-171 | 77.11% |
| CqbZIP17 | ABI5/AT2G36270 | 239.0 | 239.0 | 0.87 | 3.00E-74 | 44.09% |
| CqbZIP61 | HY5/ AT5G11260 | 193.0 | 193.0 | 1.00 | 4.00E-63 | 67.93% |
