# Supplementary material for: Genome-wide survey, characterization, and expression analysis of bZIP transcription factors in Chenopodium quinoa
Source: BMC Plant Biol. 2020 Sep 1;20:405. doi: 10.1186/s12870-020-02620-z (PMC7466520; doi:10.1186/s12870-020-02620-z)
Supplement: Supplementary file 10 — Additional file 10. Expression patterns of some duplicated CqbZIP genes in different organs and in roots under salt stress treatment. [file 12870_2020_2620_MOESM10_ESM.pdf]

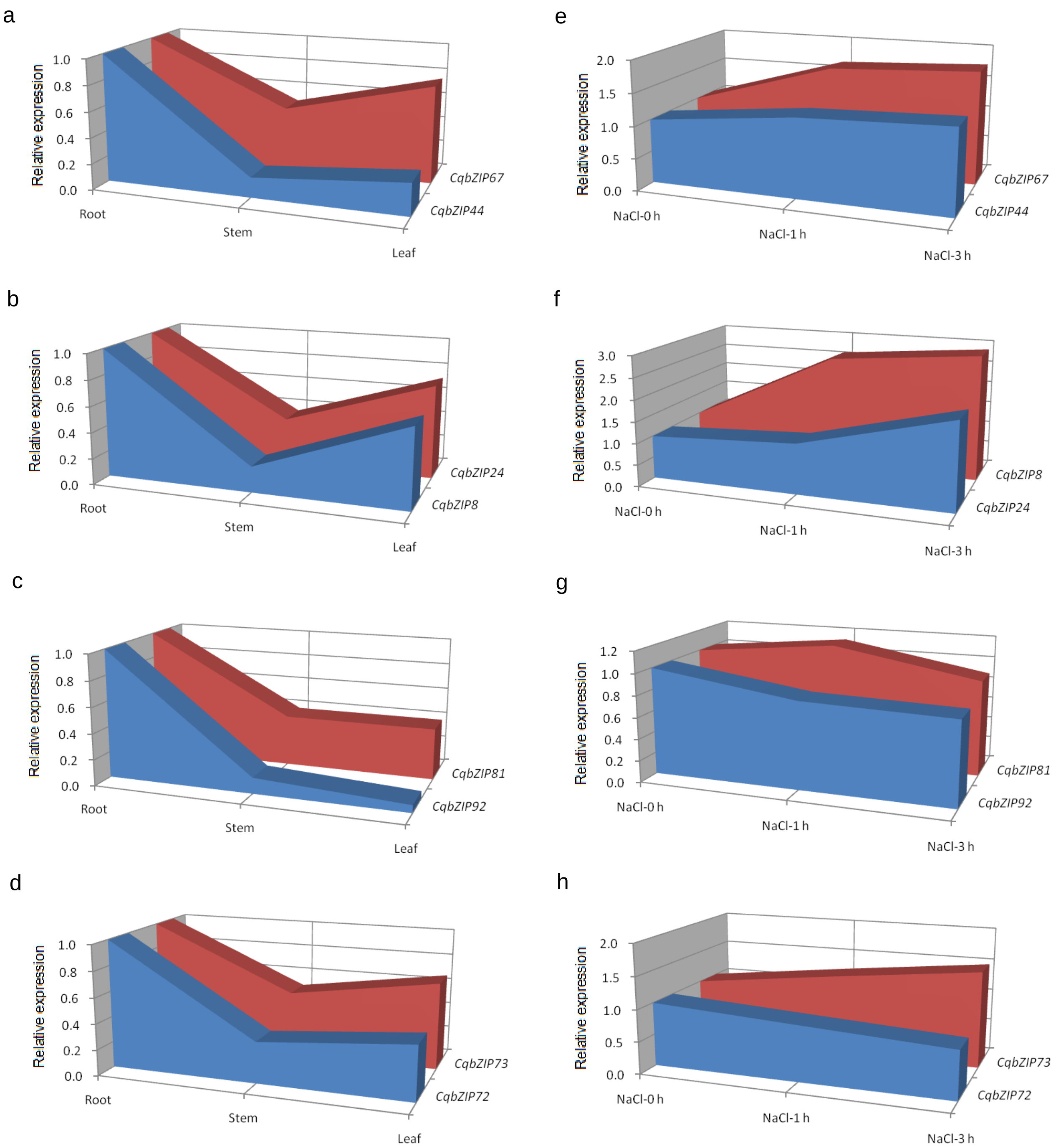

Additional file 10: Expression patterns of some duplicated *CqbZIP* genes in different organs (**a–d**) and in roots under salt stress treatment (**e–h**).
