# Supplementary material for: Genome-wide survey, characterization, and expression analysis of bZIP transcription factors in Chenopodium quinoa
Source: BMC Plant Biol. 2020 Sep 1;20:405. doi: 10.1186/s12870-020-02620-z (PMC7466520; doi:10.1186/s12870-020-02620-z)
Supplement: Supplementary file 11 — Additional file 11. Phylogenetic relationships of the bZIPs in quinoa, spinach, sugar beet, and amaranth. [file 12870_2020_2620_MOESM11_ESM.pdf]

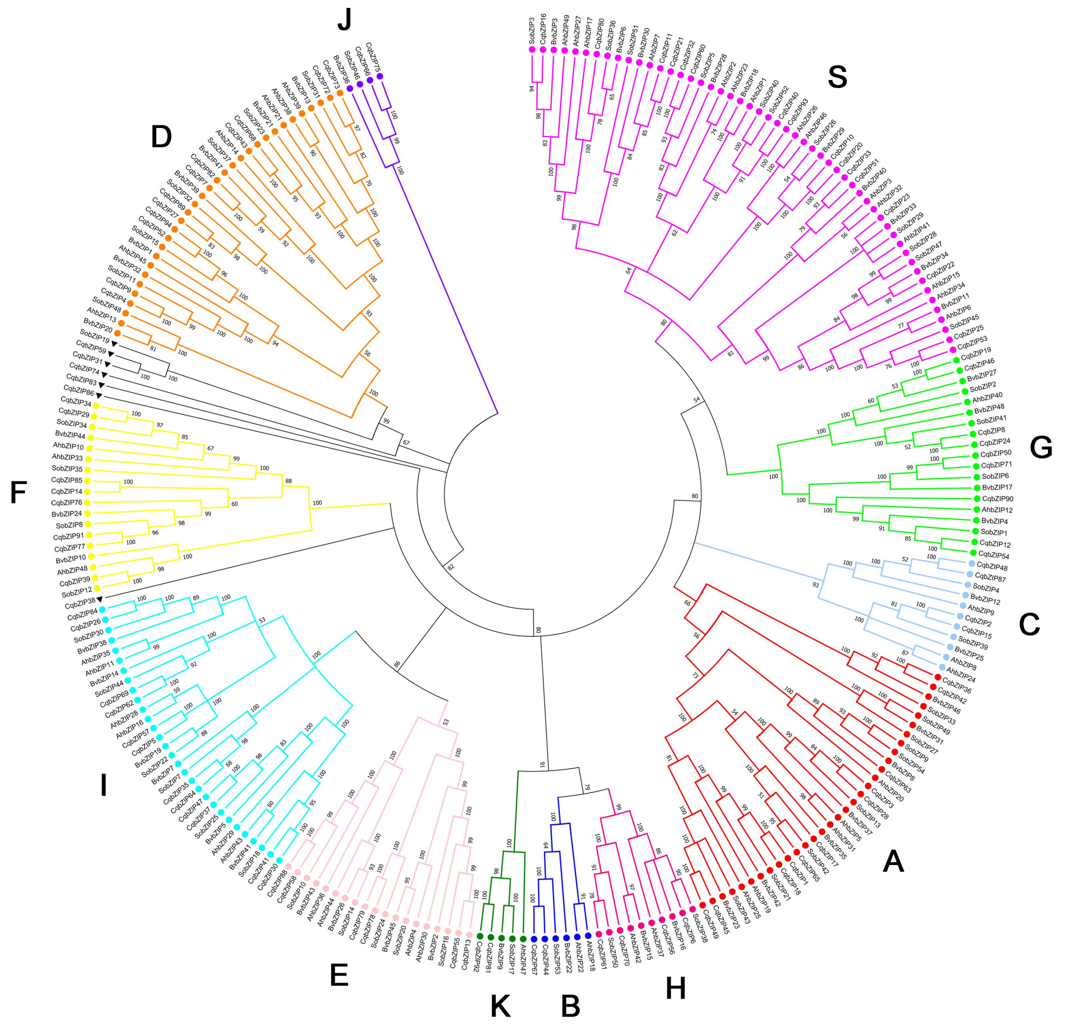

Additional file 11: Phylogenetic relationships of the bZIPs in quinoa, spinach, beet, and amaranth. Amino acid sequences were aligned using ClustalX and the neighbor-joining (NJ) tree was generated through the MEGA7 program. The subfamilies are labeled and denoted by different colors and the numbers at nodes represent bootstrap support values from 1000 replicates.
