# Supplementary material for: Genome-wide survey, characterization, and expression analysis of bZIP transcription factors in Chenopodium quinoa
Source: BMC Plant Biol. 2020 Sep 1;20:405. doi: 10.1186/s12870-020-02620-z (PMC7466520; doi:10.1186/s12870-020-02620-z)
Supplement: Supplementary file 12 — Additional file 12. PCR primers used for qRT-PCR in this study. [file 12870_2020_2620_MOESM12_ESM.doc]

**Additional file 12:** PCR primers used for qRT-PCR in this study

| Gene name | Forward primer (5'-3') | Reverse primer (5'-3') |
| --- | --- | --- |
| *EF1α* | GGTATTGGAACGGTGCCAGT | GGACTCGTGGTGCATCTCAA |
| *CqbZIP3* | GGTGCAGCTCAACAAGGTGG | CAAGCCCATCGGATGACAAA |
| *CqbZIP8* | GGATGTAAAAGATCAGAATTC | TCTCCCTGTATTGAACTAGA |
| *CqbZIP17* | TGGTGGAGCTTTCACACCTG | ATGTCCATCCCATACTGGCC |
| *CqbZIP24* | CTTTGTTGTGCTGTTGATCTAG | CTATTCTGTGCATTAGCCCC |
| *CqbZIP44* | GGCTCTAGCTCTTCTGAAGG | GCTTTGCTGCTTAGCAGTAG |
| *CqbZIP61* | TGGTTCTGCCCTTCAACTTG | CGGAAGCCGAATTTACTCCT |
| *CqbZIP67* | AGCAATAATTGGAGACACCCTCAC | CAGCATAGGCCCTGCAAGTC |
| *CqbZIP72* | AGAAAAAAACTGGAGAGTAGC | CATATTCTACATCAAAGGCC |
| *CqbZIP73* | TTCGAGTGACAGATCCAAGGAG | CTCCAGCTGTTGCACATATGC |
| *CqbZIP81* | CCGAGGATAATGTTGCTAAG | ATCAGTGTCATCATCGAGGT |
| *CqbZIP92* | GTTCCTGCAGTTCGCTCCCT | GATTTTTGCAGGCGCAGTTG |
